# Semi-Active Suspension Systems: From Advanced Control Algorithms to Emerging Off-Road and Agricultural Applications

**DOI:** 10.3390/s26154736

**Published:** 2026-07-26

**Authors:** Weidong Jia, Kangping Sun, Xiang Dong

**Affiliations:** 1 School of Agricultural Engineering, Jiangsu University, Zhenjiang 212013, China; 2212416023@stmail.ujs.edu.cn (K.S.); dongxiang@ujs.edu.cn (X.D.); 2 High-Tech Key Laboratory of Agricultural Equipment and Intelligence of Jiangsu Province, Jiangsu University, Zhenjiang 212013, China

**Keywords:** vibration isolation, magnetorheological fluid, chassis dynamics, whole-body vibration, intelligent control, terrain adaptation, ride comfort

## Abstract

Semi-active suspension systems combine low power consumption, rapid response, and fail-safe operation by reverting to passive mode after control failure, making them important for intelligent chassis and vibration-control systems. With the development of intelligent actuators, nonlinear modeling, and advanced control methods, this technology is expanding from conventional road vehicles to off-road vehicles and agricultural machinery. Compared with passenger cars, agricultural machinery faces stronger random excitation, time-varying loads, muddy environments, resource-constrained controllers, and requirements for operational accuracy. This review focuses on semi-active damping and vibration-isolation systems for off-road and agricultural applications. Mainstream actuators, control-oriented nonlinear damper models, classical, robust, and adaptive control methods, MPC, DRL, and mechanism–data fusion control are compared in terms of hardware constraints, model accuracy, real-time computation, and agricultural adaptability. Applications in seat/cab isolation, tractor and tracked chassis systems, rollover prevention, and precision implements are summarized. The review shows that semi-active suspension in agricultural machinery is evolving beyond the conventional trade-off between ride comfort and handling stability toward multi-objective coordination of safety, ground-contact stability, operational accuracy, operator protection, and energy consumption.

## 1. Introduction

The vehicle suspension system is a key chassis component that connects the vehicle body with the road surface and transmits and regulates vertical dynamic responses. Conventional passive suspension is usually designed by balancing ride comfort and handling stability: lower stiffness and damping improve ride comfort, whereas higher stiffness and damping enhance tire ground contact and vehicle handling stability [1]. Active suspension can suppress vibration over a wide frequency range through external energy input. However, its high manufacturing cost, complex hydraulic or pneumatic actuation and piping, and continuous energy demand limit large-scale application in heavy commercial, off-road, and agricultural vehicles [2]. In contrast, semi-active suspension realizes vibration control by adjusting damping or stiffness parameters. It offers lower power consumption, faster response, and a relatively simple structure. Moreover, such systems can usually revert to passive mode in the event of control failure, thereby providing good fail-safe capability. Semi-active suspension has, therefore, become an important research direction in intelligent chassis and vehicle vibration-control systems [3].

In recent years, research on semi-active suspension has shifted from early studies dominated by Skyhook control and passenger-car ride-comfort improvement toward model predictive control (MPC), deep reinforcement learning (DRL), road-preview control, and complex application scenarios such as off-road vehicles and agricultural machinery. Compared with structured on-road conditions, off-road vehicles and agricultural machinery face stronger random excitations, time-varying loads, muddy and wet environments, resource-constrained control platforms, and operational accuracy constraints. Therefore, summarizing semi-active suspension technology only from the perspective of passenger-car ride-comfort optimization is no longer sufficient to cover its engineering requirements in special vehicles for complex terrain and intelligent agricultural equipment.

Recent full-text reviews provide strong foundations but remain segmented by vehicle type or technical theme. Learning-based suspension reviews emphasize road vehicles and adaptive or reinforcement-learning methods, while also identifying limited semi-active implementation and hardware validation [2]. Automotive magnetorheological (MR) damper reviews synthesize damper structures and classical-to-intelligent control, whereas road-vehicle active-suspension reviews emphasize production hardware, packaging, power, cost, and reliability [4,5]. Preview-control reviews critically analyze sensing, preview horizon, actuator bandwidth, and MPC implementation, but do not systematically cover farmland sensing failures or implement coupling [6]. The agricultural review literature is more application-specific and concentrates mainly on operator-seat suspensions [3]. Consequently, an integrated review spanning actuator durability, high-fidelity to online modeling, control deployment, chassis/seat/implement applications, and field validation under harsh agricultural conditions remains necessary.

**Figure 1:**
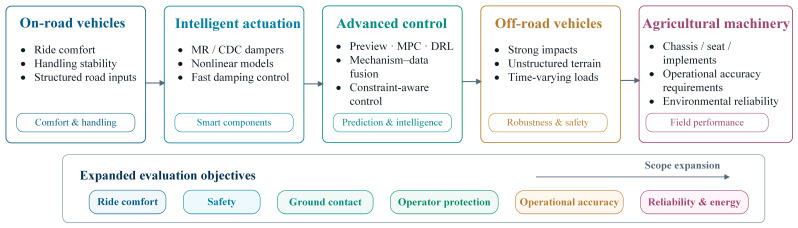
Evolution of semi-active suspension research from on-road vehicles to off-road and intelligent agricultural machinery. The scope expands from ride comfort and handling objectives to coordinated safety, ground-contact stability, operator protection, operational accuracy, reliability, and energy consumption.

The comparison in Table 1 clarifies five contributions of this review. First, it connects the technological trajectory from on-road vehicles to off-road and agricultural machinery. Second, it integrates actuator hardware, nonlinear models, high-fidelity analysis, online control, and validation rather than treating them as independent topics. Third, it evaluates agricultural constraints including dust, mud, temperature, soil–track interaction, abrupt loads, and resource-limited controllers. Fourth, it extends the objective set from ride comfort to ground-contact stability, rollover safety, operator protection, operational accuracy, reliability, and energy use. Fifth, it proposes an engineering route from high-fidelity boundary identification to reduced-order control and field verification. The resulting evolution from on-road comfort-oriented suspension research to off-road and agricultural multi-objective control is summarized in Figure 1.

To address these gaps, this review focuses on semi-active damping and vibration-isolation systems. It evaluates their development from the perspectives of intelligent actuators, dynamic modeling, advanced control algorithms, off-road and agricultural applications, and engineering deployment. Unlike traditional reviews that focus mainly on passenger-car ride-comfort optimization, this paper emphasizes the applicability and limitations of semi-active suspension under complex terrain, strong load disturbance, resource-constrained control platforms, and requirements for operational accuracy in agricultural operations. The aim is to provide a technical reference for chassis adaptive control, reduced-order structural modeling, multi-domain coordinated control, and the engineering deployment of next-generation off-road and agricultural vehicles. It also clarifies the technological pathway through which semi-active suspension is extending from road vehicles to off-road and agricultural applications.

It should be noted that the active steering, active leveling, and anti-rollover safety devices discussed in this review are briefly analyzed mainly as background technologies for chassis cooperative control or safety-boundary extension, rather than as primary semi-active suspension technologies. The focus of this review remains on semi-active dampers, semi-active seat suspensions, semi-active chassis vibration-isolation systems, and their modeling, control, and application under complex off-road conditions.

The remainder of this review is organized as follows. Section 2 reviews semi-active actuators, nonlinear damper models, environmental reliability, and the transition from high-fidelity analysis to reduced-order online models. Section 3 critically compares classical, robust, adaptive, predictive, and data-driven control, including preview, delay compensation, and cooperative control. Section 4 synthesizes off-road and agricultural applications with quantitative evidence and validation maturity. Section 5 discusses evaluation standards, real-time deployment, sensor robustness, reliability, energy, and future research priorities. Section 6 summarizes the main engineering conclusions and unresolved scientific gaps.

## 2. Intelligent Actuators and Control-Oriented Dynamic Modeling

The control performance of a semi-active suspension system is first limited by the physical capability of the underlying actuator, including maximum damping force, response bandwidth, adjustable range, energy consumption, thermal stability, and environmental durability. At the same time, stable operation of the control algorithm depends on dynamic models that can be used for real-time computation. Therefore, this section is organized around two aspects: hardware actuation capability and control-model availability. First, the structural characteristics and farmland-condition adaptability of mainstream semi-active actuators are compared. Second, differences among nonlinear damper models in fitting accuracy, parameter identification, and real-time computation are reviewed. Finally, the relationship between high-fidelity vehicle/chassis models and online reduced-order control models is discussed, laying a foundation for subsequent control strategies and engineering deployment.

### 2.1. Semi-Active Actuators and Their Hardware Constraints

In a semi-active suspension system, the actuator is the core link between control commands and physical vibration-reduction effects. Unlike active suspension, which relies on external energy to directly apply control forces, semi-active actuators usually regulate system responses by changing damping, equivalent stiffness, or energy-dissipation paths. Therefore, their performance depends not only on the control algorithm but is also constrained by hardware factors such as the damping medium, valve structure, electromagnetic response, sealing reliability, and power-supply conditions. Especially in off-road vehicles and agricultural machinery, actuators must endure low-speed large-amplitude impacts, mud and water erosion, dust contamination, temperature fluctuations, and abrupt load changes over long service periods. Their engineering applicability should, therefore, not be evaluated solely on the basis of maximum damping force or laboratory-measured response time.

Currently, widely used semi-active actuators include magnetorheological (MR) dampers, continuous damping control (CDC) dampers, self-powered/energy-regenerative dampers, and quasi-zero-stiffness or bio-inspired vibration-isolation structures. These actuators differ significantly in response speed, packaging space, cost, energy consumption, and durability in harsh environments. For comparison, Table 2 summarizes the response capability, power demand, environmental durability, maintenance requirements, validation maturity, and agricultural suitability of mainstream semi-active actuators and vibration-isolation devices. Because the reviewed studies used different actuator architectures, loading conditions, excitations, temperatures, and validation procedures, direct cross-study ranking is not appropriate. Table 2, therefore, separates reported performance from author interpretation. It also includes available test-exposure evidence and marks unavailable quantities as not reported (NR).

The evidence summarized in Table 2 remains uneven across technologies. Linear and hybrid MR dampers provide directly reported hardware-response and sealing-test evidence, whereas rotary MR, CDC, regenerative, and QZS studies mainly provide technology-specific prototype, simulation, or laboratory results. Standardized agricultural service-life data, endurance cycles, maintenance intervals, long-term temperature-dependent attenuation, and multi-season field validation remain largely unreported. The NR entries, therefore, identify evidence gaps rather than implying poor technology performance, and the agricultural interpretations should be understood as author synthesis rather than direct cross-study rankings.

Within the reported test conditions, linear MR dampers have demonstrated rapid force regulation and broad adjustable damping. However, axial installation space, sealing reliability, coil heating, fluid sedimentation, and temperature-dependent force attenuation remain relevant constraints for long-term agricultural operation [8,14,15]. Rotary MR dampers provide compact packaging and have demonstrated prototype- and seat-level vibration-control potential, but agricultural endurance, contamination resistance, and maintenance requirements remain insufficiently reported [9,10].

The reviewed CDC studies mainly demonstrate continuously adjustable hydraulic damping through bench-informed modeling and vehicle-level simulation. One variable-domain fuzzy PID study reported simulated reductions of 49.6%, 50%, and 50% in the peak suspension dynamic deflection, body acceleration, and dynamic load, respectively. However, harmonized actuator-response time, electrical-power demand, valve or seal endurance, temperature-dependent force attenuation, maintenance intervals, and agricultural field validation were not reported [11]. Accordingly, CDC dampers remain a potentially serviceable hydraulic route, but their superiority over MR dampers cannot be established from the available non-harmonized evidence.

Regenerative and self-powered dampers combine vibration attenuation with energy harvesting, but their reported performance depends strongly on the specific architecture. One integrated self-powered MR-damper prototype reported a maximum damping force of 4156 N, a dynamically adjustable range of 6.25, a maximum instantaneous recovered power of 29.1 W, and an energy-recovery efficiency of 11.2–24.6% [12]. These results demonstrate prototype-level feasibility rather than long-term system-level energy benefit; service life, endurance cycles, average recovered power under agricultural duty cycles, maintenance intervals, and long-term field validation remain unreported. Other self-sensing and energy-harvesting architectures broaden the design space but cannot yet be directly ranked under common test conditions [16,17].

QZS and bio-inspired isolators provide a potential route for local low-frequency vibration isolation. A trapezoidal-beam QZS prototype reported an initial isolation frequency of 2.91 Hz and was evaluated through finite-element analysis, static compression testing, and laboratory dynamic experiments [13]. However, structural endurance, environmental resistance, maintenance intervals, payload-dependent performance boundaries, and vehicle or agricultural field validation remain insufficiently reported. MR-assisted and bio-inspired variants indicate further design potential, but the available evidence remains predominantly prototype- or laboratory-based [18,19].

Overall, the available evidence supports technology-specific application potential rather than a universal performance ranking. MR and CDC dampers appear to be plausible near-term candidates because of their existing component- and vehicle-level evidence, but this interpretation remains an author synthesis and requires confirmation through harmonized agricultural endurance, thermal, maintenance, and multi-season field studies. Regenerative devices are promising where auxiliary sensing power is valuable, whereas QZS structures are more directly suited to seat, cab, or implement-level low-frequency isolation.

Environmental reliability must be treated as a separate design dimension rather than being hidden within nominal damping performance. Dynamic-seal tests show that solid particles in MR fluid can promote abrasive wear of O-rings; particle size and mass fraction increase friction and wear risk, while the reported test evidence does not yet establish a standardized farmland service life [7]. Vehicle-level MR-damper experiments demonstrate approximately 10 ms hardware response for a hybrid valve architecture and 30–40% reductions in selected body-motion peaks under specific road-vehicle maneuvers, but these values are not complete closed-loop delays and should not be transferred directly to agricultural duty cycles [8]. Moreover, probabilistic calibration of MR model parameters is needed because parameter uncertainty propagates into suspension responses; such reliability analysis concerns model uncertainty rather than mechanical service life [20]. Therefore, future actuator qualification should report sealing condition, abrasive contamination, temperature cycle, force attenuation, maintenance intervention, and validation duration.

### 2.2. Nonlinear Damper Models for Real-Time Control

Semi-active actuators exhibit significant nonlinear characteristics, especially hysteresis, saturation, force lag, temperature drift, and electromagnetic response delay in MR dampers. These nonlinear factors mean that controllers cannot rely only on simple linear damping models; otherwise, damping-force prediction errors are likely under high-frequency impacts or large-amplitude excitations. For semi-active suspension, the dynamic model must satisfy two requirements. On the one hand, it should provide sufficient fitting accuracy to describe the force–velocity, force–displacement, and current–damping force relationships of the damper. On the other hand, it must have low computational complexity to meet the real-time control requirements of vehicle or agricultural machinery ECUs. Therefore, the core issue in nonlinear damper modeling is not the pursuit of maximum fitting accuracy alone, but the balance among model accuracy, parameter identification difficulty, invertibility, and online computational cost.

To address these issues, common semi-active damper models currently include the modified Bouc–Wen model, the Prandtl–Ishlinskii (PI) model, algebraic/magic-formula models, and Hammerstein or neural network cascade models. Table 3 compares the identification requirements, nonlinear representation capability, online computational burden, validation maturity, and engineering application boundaries of representative nonlinear damper models.

As shown in Table 3, different nonlinear damper models differ significantly in fitting accuracy, parameter identification, and real-time deployment. The modified Bouc–Wen model has strong hysteresis-description capability and is suitable for offline modeling, simulation analysis, and controller validation. However, it contains many parameters and is difficult to invert online, making it unsuitable as a real-time model for low-cost agricultural machinery ECUs [21,22]. The Prandtl–Ishlinskii (PI) model has a clear structure, low computational burden, and analytical invertibility, making it more suitable for low-delay damping-force tracking. Nevertheless, under temperature drift, fluid inertia, and strong force-lag conditions, it still needs to be combined with online correction or piecewise modeling [26].

Algebraic/magic-formula models have simple forms and low computational cost, making them suitable for embedding into MPC, fuzzy control, or real-time optimization frameworks. However, their accuracy depends on the range of experimental calibration, and errors may increase outside the calibrated conditions [23,27]. Gray-box or data-driven models such as Hammerstein and neural network models can approximate complex hysteresis, fluid inertia, and force lag, but they depend on high-quality training data and require lightweight design and physical-constraint assistance on resource-constrained platforms [28,29]. Therefore, a reasonable route is to use high-accuracy models for offline calibration and error analysis, and then simplify them into PI models, piecewise algebraic models, or other invertible low-order models for online control.

**Figure 2:**
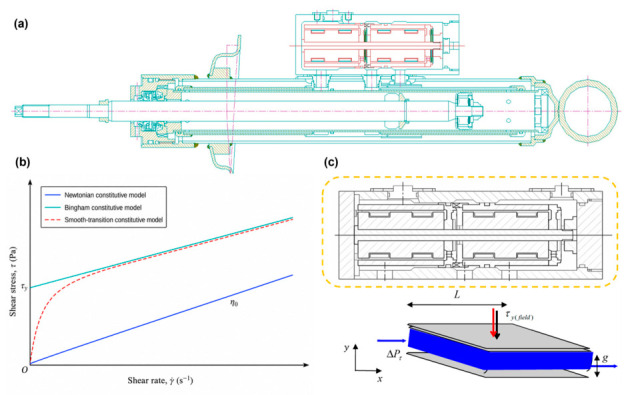
Structural, rheological, and pressure-drop mechanisms governing the controllable force of an external-valve magnetorheological (MR) damper: (**a**) overall assembly of the MR damper with an external MR valve; (**b**) comparison of the Newtonian, Bingham, and smooth-transition constitutive models, where the vertical and horizontal axes denote shear stress and shear rate, respectively; and (**c**) valve-level pressure-drop mechanism induced by MR-fluid flow, with an inset showing the cross-sectional structure of the external MR valve. Together, these mechanisms explain how damper architecture, field-dependent rheology, and valve pressure drop determine the achievable damping-force range and dynamic-response boundary.

In addition to macroscopic hysteresis modeling, the attainable damping-force boundary of an MR damper is fundamentally governed by its structural configuration, field-dependent rheological behavior, and valve-level pressure-drop mechanism. As summarized in Figure 2, the external-valve arrangement defines the principal fluid-flow path and magnetic activation region, thereby providing the structural basis for controllable resistance generation [8]. At the material level, the rheological response of MR fluids can be represented by Newtonian, Bingham, and smooth-transition constitutive relationships. The Bingham model captures the essential field-dependent yield-stress behavior, whereas the smooth-transition formulation removes the abrupt discontinuity near zero shear rate and is, therefore, more suitable for numerical simulation and control-oriented modeling [29]. At the valve level, the field-induced yield stress generates an additional pressure drop across the activated flow channel, which is subsequently converted into a controllable damping force [8]. These structural, rheological, and fluid-dynamic mechanisms jointly determine the achievable force range, response characteristics, and practical control constraints of MR dampers. Although detailed electromagnetic and fluid-dynamic representations are valuable for actuator design and damping-boundary identification, their computational complexity generally prevents their direct implementation in real-time suspension controllers.

### 2.3. High-Fidelity Modeling and Reduced-Order Representation

Semi-active suspension control depends not only on damper models, but also on the overall dynamic characteristics of the vehicle or agricultural machinery chassis. For structured road vehicles, quarter-car, half-car, and full-vehicle seven-degree-of-freedom models are commonly used for controller design and performance evaluation. However, in off-road vehicles and agricultural machinery, vehicle–ground interaction, track engagement, multi-source implement excitation, frame flexibility, abrupt load changes, and cab/seat coupling are more complex. Traditional low-order vehicle models often fail to fully describe real system responses. Therefore, before advanced control strategies are introduced, high-fidelity modeling is needed to identify structural modes, vibration transmission paths, and physical safety boundaries.

High-fidelity multibody dynamics, finite-element models, and modal tests are commonly used for chassis and frame-structure analysis of agricultural equipment. For example, rigid-flexible coupled modeling based on SolidWorks 2020 and RecurDyn V9R5 can be used to analyze dynamic stress concentration regions in the leveling chassis of tracked harvesters. For rice combine harvester frames, comparison between a seven-degree-of-freedom dynamic model under multi-source excitation and measured vibration responses has shown that combined modal frequencies of the chassis frame and threshing frame may fall within the operating excitation-frequency range, thereby inducing complex vibration coupling and resonance. Modal and harmonic-response analyses based on Ansys Workbench are also often used to study frequency-domain responses and vibro-acoustic transmission paths in orchard weeder chassis, combine harvester conveying troughs, vibrating-screen bolts, and support structures of threshing devices [30,31,32,33,34,35,36]. These models help researchers identify structural resonance, local stress concentration, flexible-connection effects, and implement-excitation transmission paths that are difficult for low-order control models to capture, thereby providing a basis for reduced-order modeling, safety-constraint setting, and structural damping optimization in suspension controllers.

For hilly, mountainous, and tracked agricultural equipment, full-vehicle dynamic modeling also needs to consider coupling among pitch, roll, vertical vibration, track–soil contact, and implement disturbance. For example, hilly chassis can exhibit obvious pitch-roll coupled vibration on unstructured roads [37]. Attitude compensation of wheel-legged chassis needs to combine zero-moment-point theory with multibody dynamics modeling [38]. Reverse-synchronous hydraulically interconnected suspension for three-axle heavy trucks and transverse leaf-spring systems also require hydro-mechanical coupled or multibody dynamic models to describe load transfer and medium- to high-frequency vibration characteristics [39,40]. In conventional tractors, high-dimensional models including the vehicle body, cab, seat, and human body help reveal whole-body vibration transmission paths and provide a basis for parameter optimization of seat suspension and cab mounts [41,42,43].

In more complex soil–track interaction scenarios, a coupled discrete-element method–flexible multibody dynamics (DEM-FMBD) analysis framework can more realistically describe track obstacle crossing, soft-soil contact, and dynamic stress distribution [44]. Similarly, multi-source vibration modeling of combine harvester headers, tractor-rotary-tiller units, and frame shell structures can explain broadband vibration problems caused jointly by cutting, conveying, screening, and ground excitation during agricultural machinery operation [45,46,47]. In addition, finite-element analysis and transfer path analysis (TPA) can be used to identify the main vibration transmission paths from cutter, frame, and mounts to the driver seat, thereby supporting reduced-order design and sensor placement for semi-active suspension controllers [48,49,50,51].

It should be emphasized that high-fidelity models are not suitable for direct use as online control models. Although software platforms such as RecurDyn, ADAMS, and Ansys, together with coupled DEM-FMBD analysis frameworks, can accurately reproduce structural flexibility, contact nonlinearity, and multibody coupling effects, they have high degrees of freedom and heavy computational burdens, making it difficult to satisfy millisecond-level or near-real-time control requirements of semi-active suspension. Their core value lies in offline boundary identification: modal analysis, transmission-path identification, and extreme-condition simulation can be used to determine inherent frequency bands, resonance risks, maximum dynamic travel, stress-concentration regions, and allowable control boundaries of chassis structures.

**Figure 3:**
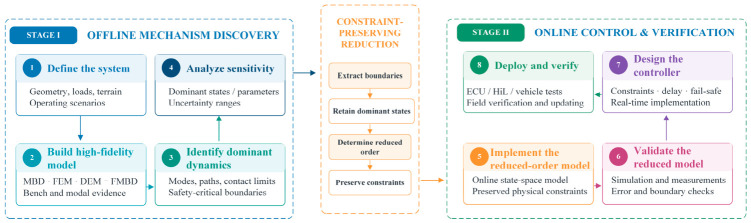
Conceptual engineering workflow linking high-fidelity agricultural-machine models to online semi-active suspension control. High-fidelity models identify dominant modes, vibration paths, contact boundaries, and safety-critical constraints; sensitivity analysis and bench evidence then support reduced-order, physics-constrained models that are validated before ECU, HiL, vehicle, and field deployment.

Therefore, semi-active suspension modeling for agricultural machinery should follow the engineering logic of using offline high-fidelity modeling to define boundaries and online reduced-order models for rapid solution. High-fidelity models are used to identify structural modes, vibration transmission paths, and ultimate safety boundaries. Actuator bench tests are used to calibrate nonlinear damping models. Online control should employ PI models, algebraic fitting models, low-order vehicle models, or lightweight data models constrained by physics. This framework avoids the lack of physical boundaries in purely black-box control and reduces the delay risk caused by direct online computation of high-dimensional models. The corresponding conceptual and constraint-preserving workflow from offline high-fidelity analysis to online suspension control is summarized in Figure 3.

Figure 3 should be interpreted as a conceptual synthesis rather than a literature-reported worked reduction case. In the agricultural studies reviewed, high-fidelity multibody, finite-element, and contact models were generally used to identify structural modes, vibration paths, stress concentrations, and contact boundaries, whereas low-order suspension and vehicle models were developed separately for controller design and state estimation. We did not identify a study that documented the entire model-reduction chain for an agricultural chassis. Missing information included the retained modes or states, preserved physical constraints, explicit reduced-order equations, and quantitative reduction errors. The absence of such a traceable end-to-end reduction example, therefore, remains an important research gap.

Section summary: The engineering performance of semi-active suspension is jointly determined by actuator physical capability and control models. For agricultural machinery and off-road vehicles, actuators must balance response speed, maximum damping, cost, energy consumption, and environmental durability, while models must balance nonlinear fitting accuracy and real-time computational complexity. Overall, MR dampers and CDC dampers appear to be plausible near-term hardware routes, although agricultural endurance and field evidence remain limited. PI models, algebraic models, and other low-order invertible models are more suitable as control-oriented online models, whereas high-fidelity multibody/finite-element models should mainly serve offline boundary identification, structural optimization, and controller validation.

## 3. Semi-Active Suspension Control Strategies

Actuators define the achievable damping limits of a semi-active suspension system. Control algorithms determine how effectively these limits are used under random excitation, actuator constraints, and competing objectives. Unlike active suspension, semi-active suspension cannot inject energy into the system arbitrarily. Its control force must satisfy passivity constraints such as damping-force direction, actuator saturation, and suspension dynamic travel. The control objective is, therefore, to coordinate ride comfort, tire/track contact, suspension travel, energy use, and component life within the actuator’s adjustable range.

According to control philosophy and deployment maturity, semi-active suspension control can be grouped into classical/robust methods, fuzzy/adaptive methods, MPC variants, data-driven methods, and mechanism–data fusion methods. Because the reviewed studies used different vehicle models, processors, sampling periods, excitations, and validation procedures, qualitative labels such as low, moderate, or high are not directly comparable unless identified as author synthesis. Table 4, therefore, distinguishes directly reported evidence from author synthesis and marks unavailable processor timing or worst-case execution time (WCET) as NR. The table is intended as an evidence-boundary comparison rather than a universal performance ranking.

The evidence summarized in Table 4 shows that processor-specific timing and WCET remain unreported for most reviewed control strategies. Explicit preview MPC was implemented on an NVIDIA Jetson AGX Xavier, but its WCET was not reported. Similarly, the approximately 2.05 ms computational response reported for a mechanism–data fusion controller was obtained on a desktop PC rather than an agricultural ECU [25,59]. Therefore, the qualitative model dependence, adaptation capability, and agricultural-deployment entries in Table 4 should be interpreted as evidence-informed author synthesis rather than harmonized processor benchmarks. Future studies should explicitly report processor type, sampling period, average and worst-case execution time, deadline-miss behavior, fail-safe logic, and HiL, vehicle, or field validation.

### 3.1. Classical, Robust, and Adaptive Control

Classical and robust control represent relatively mature engineering routes for semi-active suspension. Skyhook, Groundhook, and Hybrid control are based on simple physical rules and have the advantages of clear structure, low computational burden, and easy vehicle implementation. However, their control laws usually rely on fixed rules and have limited adaptability under unstructured terrain, abrupt load changes, and actuator hysteresis. Therefore, in complex agricultural machinery scenarios, conventional rule-based control generally needs to be combined with fuzzy logic, adaptive parameter tuning, or robust control methods to improve adaptability to strong random excitation.

Active disturbance rejection control (ADRC) estimates internal uncertainty and external disturbances through an extended state observer and has low dependence on accurate models. It is suitable for handling multi-source disturbances in agricultural machinery caused by soil unevenness, load variation, and implement excitation. Studies have applied LADRC, PIMO optimization, and ANFIS inverse models to seat/body vibration reduction and damping-force prediction, demonstrating the engineering value of ADRC in low-model-dependence and strong-disturbance compensation [52,61,62]. In addition, ADRC can be combined with dynamic inerter suspension or LMI output-feedback control for low-frequency resonance suppression and body attitude control under load-varying conditions [63,64].

Sliding mode control (SMC) has strong robustness and is suitable for nonlinear systems and parameter uncertainties. In agricultural machinery path tracking, chassis leveling, and suspension vibration reduction, adaptive SMC, fuzzy SMC, and extended state observers are commonly used to suppress paddy-field slip errors, attitude disturbances, and vertical vibration [65,66,67]. However, the high-frequency switching of SMC may cause chattering and increase wear in valves, coils, or mechanical connectors. In practical applications, smooth switching functions, fractional-order sliding modes, ANFIS inverse models, or observer-assisted strategies are, therefore, often used to reduce chattering and improve nonlinear tracking capability [54,55,68,69].

Fuzzy control and adaptive control are more suitable for handling parameter drift, model uncertainty, and changing operating conditions. Interval type-2 fuzzy control, nonlinear fuzzy PI-PD, variable-universe fuzzy PID, and fuzzy sliding mode control can improve the adaptability of semi-active suspension to time-varying conditions through rule bases and online parameter adjustment. In height-adjustment systems of electronically controlled air suspension for agricultural vehicles, single-neuron adaptive PID also reflects an engineering basis for low-cost control [11,56,70,71,72]. To reduce dependence on manual tuning, swarm-intelligence optimization methods such as DBO, MFWA, and GA are often used for fuzzy-controller parameter optimization [73,74,75,76,77].

Overall, classical, robust, and adaptive control have advantages such as low computational burden, strong structural interpretability, and low engineering implementation cost, and remain realistic near-term control routes for low-cost agricultural machinery platforms. However, their parameter tuning depends heavily on experience. Under strongly time-varying soil conditions, complex implement disturbances, and multi-objective constraints, they still need to be combined with constraint optimization, preview perception, or data-assisted methods.

### 3.2. Model Predictive Control and Constraint Handling

The core advantage of model predictive control (MPC) is its ability to simultaneously handle system models, control objectives, and constraints within a receding-horizon optimization framework. For semi-active suspension, maximum actuator damping force, damping-force direction, suspension dynamic travel, tire/track ground contact, and body attitude response can all be incorporated into objective functions or constraints in a unified design. Therefore, the value of MPC lies not only in improving vibration-reduction performance, but also in explicitly coordinating conflicts among ride comfort, handling stability, ground-contact stability, energy consumption, and hardware actuation frequency.

In vehicle suspension research, MPC has been used to coordinate body acceleration, suspension dynamic travel, and dynamic tire load. GA-MPC can reduce dynamic wheel load and decrease high-frequency actuation of proportional valves. MLD-MPC and MIQP frameworks can describe the nonlinear and switching characteristics of semi-active suspension. MLD-based MPC can also be extended to body attitude control and rollover-risk suppression [57,78,79]. These studies indicate that MPC is more suitable for semi-active suspension problems with clear constraint boundaries and multi-objective conflicts.

The main obstacles to applying MPC in agricultural machinery are computational load and model accuracy. Agricultural machinery controllers are usually deployed on low-cost ECUs, while vehicle models are affected by soil stiffness, track contact, load variation, and implement disturbance. Directly using long prediction horizons, high-dimensional models, or complex nonlinear optimization can easily lead to excessively long control cycles. To reduce real-time computational pressure, IHOS-MPC, explicit MPC, VHMPC, and MPC-PID fusion control improve engineering deployability through prediction-horizon compression, offline explicit solution, adaptive prediction windows, and hierarchical inner/outer-loop compensation, respectively [59,80,81,82]. For special off-road vehicles, adaptive MPC combined with state observers has also demonstrated preliminary feasibility in HiL validation [58]. Explicit MPC shifts online optimization to offline multiparametric partitioning and online table lookup. In a full-scale all-terrain vehicle, preview explicit MPC was deployed on an NVIDIA Jetson AGX Xavier. Relative to passive suspension, it reduced measured seat-weighted acceleration RMS by 29.6% and pitch and roll acceleration RMS by 24.1% and 26.7%, respectively. However, the study did not report worst-case controller execution time, and the results are specific to its road-test configuration [59].

MPC for agricultural machinery should not directly copy high-dimensional receding-horizon optimization frameworks developed for passenger cars. Instead, it should be combined with reduced-order actuator models, offline high-fidelity boundary identification, and explicit constraint sets. In sprayers, seeders, and hilly tractors, control objectives may simultaneously include operator comfort, implement attitude, track/tire ground contact, roll safety, and energy constraints. MPC is suitable for constructing such a multi-objective optimization framework, provided that the model is sufficiently simple, the constraints are clearly defined, and the control platform can satisfy real-time requirements.

### 3.3. Data-Driven and Mechanism–Data Fusion Control

Data-driven methods provide new control ideas for strong nonlinearity, complex random excitation, and soil–vehicle interactions that are difficult to model accurately in semi-active suspension. DRL can learn control strategies through interaction with the environment and avoid complete dependence on manually designed rules. Algorithms such as DDPG, PPO, and TD3 have been used in semi-active or active suspension control to improve adaptability under complex excitation and tolerance to parameter perturbations. Methods combined with NODE can also be used to describe continuous dynamic characteristics of MR dampers [24,60,83].

Neural networks more often serve as auxiliary modules for traditional controllers rather than complete replacements for physical control laws. Methods such as RBFNN, ANN, LSTM, and EKF can compensate for unmodeled dynamics, tune control parameters online, or predict agricultural machinery operating states, thereby providing feedforward information for semi-active suspension [84,85,86]. This route may offer a more interpretable and engineering-oriented alternative to pure DRL because it retains the main controller structure and physical constraints, making engineering interpretation and safety validation easier.

Although data-driven methods have potential, they still face obvious barriers in real-vehicle deployment for agricultural machinery. First, pure DRL strategies are black-box in nature, making it difficult to explain the causal relationship between control actions and vehicle dynamic responses. Second, field data collection is costly, operating conditions have poor repeatability, and extremely dangerous conditions are difficult to cover. Third, if the reward function is poorly designed, the controller may reduce body acceleration at the expense of suspension dynamic travel, ground contact, or rollover-prevention safety boundaries. Therefore, some studies have introduced physical constraints such as maximum dynamic travel into DDPG reward mechanisms to limit out-of-bounds control actions and improve safety [87]. This indicates that DRL control for agricultural machinery must be combined with physical boundaries and engineering validation. A representative mechanism–data fusion study, described by its authors as a mechanism–data-driven control strategy, reported a computational response time of approximately 2.05 ms for its trained controller on a standard PC (Intel Core i9-12900KF, 16 GB RAM). However, HiL and vehicle-level validation remain outstanding, and this value should not be interpreted as a certified agricultural-ECU execution time or WCET [25]. Likewise, learning-based control must report network size, sampling period, worst-case execution time, training coverage, and fail-safe behavior before field deployment.

**Figure 4:**
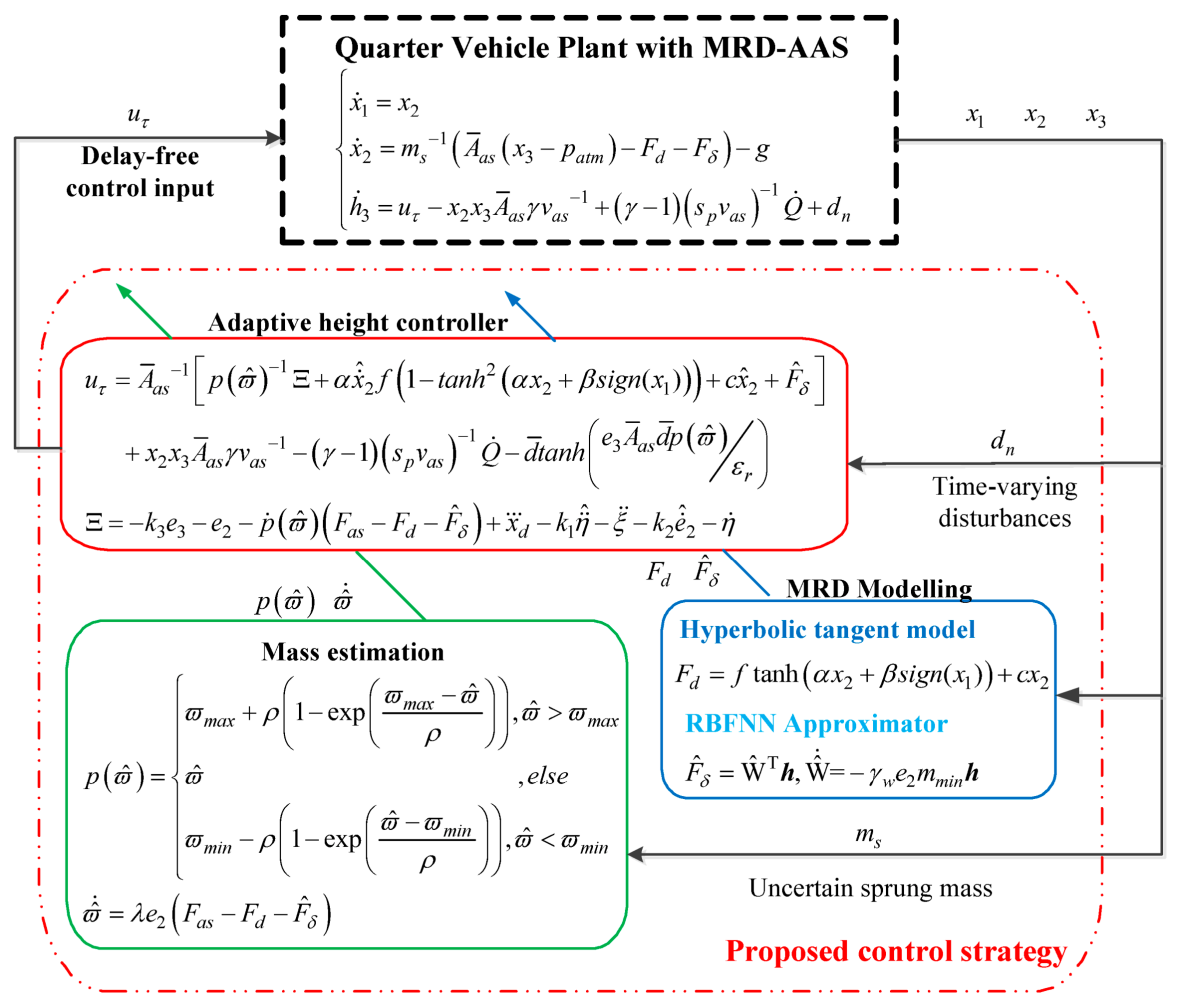
Representative mechanism–data fusion adaptive control architecture for an MR-damper-assisted active air suspension system. The framework integrates measured suspension states, a physics-based plant model, RBF neural network compensation of unmodeled MR-damper dynamics, projection-based estimation of uncertain sprung mass, and time-delay compensation to improve robustness under parameter uncertainty and delayed actuation.

In this review, mechanism–data fusion control refers to architectures that retain an explicit physical or mechanism-based model while using data-driven modules for residual compensation, parameter estimation, or policy improvement. Such architectures provide a more defensible route for overcoming the black-box limitations of purely data-driven methods. One representative route uses differential-geometry-based vehicle dynamics to construct the main control framework and employs TD3 reinforcement learning to compensate for nonlinear residual dynamics, thereby combining physical interpretability with data-driven adaptability [25]. A complementary mechanism–data fusion route retains the main suspension and actuator dynamics while introducing a neural network module only for uncertain or unmodeled components. As illustrated in Figure 4, Zhao et al. developed a physics-based adaptive control architecture for an MR-damper-assisted active air suspension. An RBF neural network approximator compensated for concentrated MRD modeling errors, a projection-based estimator addressed uncertain sprung mass, and a delay-compensation mechanism handled time-varying actuator input delay [84].

Figure 4 demonstrates a representative engineering form of mechanism–data fusion control in which the neural network does not replace the entire controller but compensates for modeling errors within a physically interpretable closed-loop structure. Compared with unconstrained end-to-end DRL, this architecture retains explicit suspension dynamics, state feedback, parameter-estimation logic, and delay compensation, which facilitates stability analysis and engineering interpretation. Nevertheless, the cited study focuses on ride-height tracking of an MR-damper-assisted active air suspension and was validated through AMESim 2021.2–MATLAB/Simulink 2019b co-simulation rather than agricultural field experiments [84]. Therefore, its direct relevance to agricultural semi-active suspension lies mainly in the transferable architecture: physical models can define actuator and safety boundaries, while lightweight data-driven modules can compensate for soil-dependent uncertainty, implement disturbances, actuator drift, and time-delay effects. Hardware-in-the-loop, vehicle-level, and field validation are still required before such approaches can be deployed on agricultural machinery.

### 3.4. Preview, Delay Compensation, and Integrated Control

Semi-active suspension control is evolving from traditional feedback vibration reduction toward perception preview, delay compensation, and multi-domain collaboration. Traditional feedback control responds only after the wheel or body has been excited, whereas preview control can obtain terrain information ahead of the vehicle and adjust damping states before impact occurs. For off-road vehicles and agricultural machinery, preview control is important not only for reducing body acceleration, but also for suppressing chassis attitude variation in advance, thereby protecting the spatial stability of operating implements such as booms, seeding units, and headers.

In terms of preview perception, binocular vision, road-profile extraction, virtual reference profiles, IMU-based state estimation, and wheelbase preview can all provide feedforward information for semi-active suspension. Vision-based preview is suitable for obtaining terrain changes at longer distances, but its reliability is limited in muddy, dusty, and occluded environments. IMU and wheelbase preview are lower in cost but have limited prediction ranges. Related studies have shown that combining road preview with MPC, fuzzy PID, or real-vehicle preview control can improve impact suppression and anti-pitch performance to a certain extent [88,89,90,91,92].

Actuator delay and phase deviation must be addressed when preview control moves toward real-vehicle application. MR dampers, CDC dampers, and hydraulically interconnected structures may all involve electromagnetic response, fluid hysteresis, or spool motion delay. When there is an obvious phase difference between the control command and the actual damping force, the control force originally intended for vibration reduction may weaken the control effect in some frequency bands. To solve this problem, robust H2, NSGA-II-TLQR, first-order Taylor expansion, dual-delay feedback optimization, and improved Smith predictors have been used to compensate for control-flow delay. Some studies also improve phase deviation through inerter suspension and mechanical impedance design [23,93,94,95,96].

**Figure 5:**
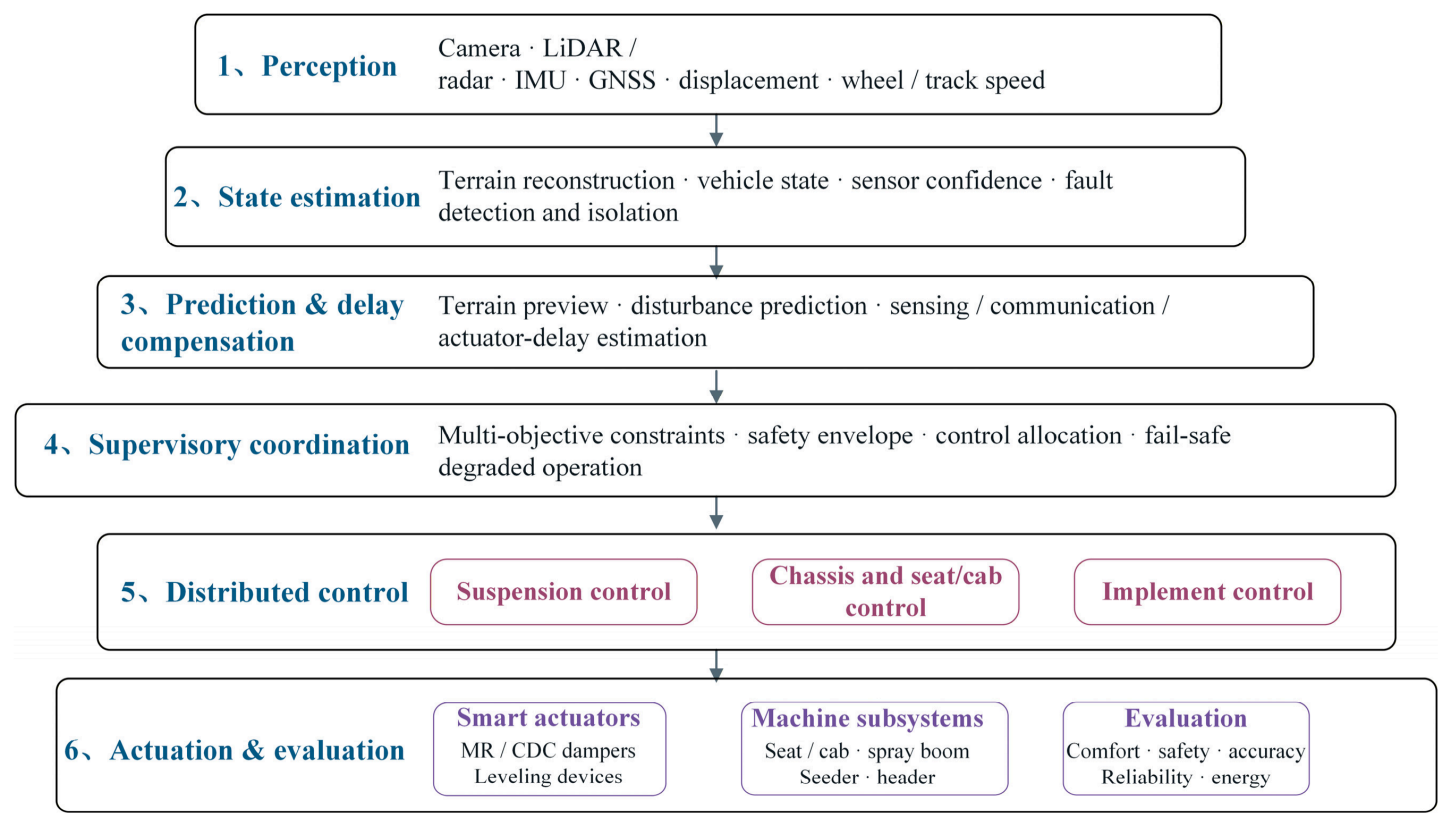
Integrated perception–delay–suspension–implement control architecture for agricultural machinery. The layered framework combines multi-source sensing, confidence-aware state estimation, terrain preview, delay compensation, supervisory constraints, distributed control, and fail-safe feedback, while decomposing end-to-end latency into sensing, estimation, communication, computation, and actuation components.

With the development of intelligent chassis technologies, semi-active suspension is no longer an isolated vertical vibration-control subsystem but is evolving toward coordinated control of suspension, steering, braking, traction, and operating implements. Representative studies have coupled longitudinal–lateral trajectory tracking with vertical suspension control by using vehicle speed and road excitation as shared variables between the control layers [97]. The broader architecture presented in Figure 5 further extends this concept by incorporating multi-source sensing, perception-confidence assessment, delay compensation, implement coordination, and fail-safe degraded control. Similar ideas can also be extended to chassis-control scenarios such as trailer attitude management, line-of-sight stabilization of autonomous-driving sensors, orchard-robot obstacle avoidance, spraying path control, torque distribution, and track-slip compensation [98,99,100,101,102,103].

It should be noted that the effectiveness of preview and collaborative control depends strongly on perception accuracy, delay compensation, and actuator response capability. Vision and LiDAR may fail under muddy, dusty, strong-light, or occluded conditions. Although multi-domain collaborative control can improve overall performance, it also increases control-architecture complexity and safety-validation difficulty. Therefore, a more realistic route for agricultural machinery is to combine low-cost preview perception, reduced-order vehicle models, constraint-based controllers, and fault-tolerant mechanisms, rather than simply pursuing high-complexity global optimal control. A field-validated VIO–MPC orchard-navigation study reported a camera rate of 30 FPS, an IMU rate of 100 Hz, and a localization latency of approximately 60 ms per image. Although it did not constitute a suspension-control loop, it illustrates the sensing and computation constraints that must be considered in preview-based suspension control [104]. Figure 5, therefore, treats latency as an end-to-end quantity and includes confidence assessment and degraded control when perception quality falls.

Section summary: Semi-active suspension control is evolving from rule-based methods toward constraint optimization, mechanism–data fusion, and multi-domain coordination. Classical, robust, and fuzzy adaptive control remain practical for resource-constrained agricultural platforms, whereas MPC is more suitable for explicit constraint handling and multi-objective optimization. DRL and neural network methods show potential under complex nonlinear conditions, but physical safety boundaries, interpretability, real-time capability, and field validation remain essential before agricultural deployment.

## 4. Off-Road and Agricultural Machinery Applications

Compared with structured road vehicles, off-road vehicles and agricultural machinery differ significantly in excitation sources, operating objectives, and safety constraints. Passenger-car suspension is usually evaluated mainly by body vertical acceleration, suspension dynamic travel, and dynamic tire load, whereas agricultural machinery must also consider soil–vehicle interaction, track or tire ground-contact stability, implement attitude, chassis rollover prevention, operator occupational health, and long-term service reliability. Therefore, when semi-active suspension is extended from passenger-car scenarios to agricultural equipment, the conventional trade-off between ride comfort and handling stability cannot simply be adopted. Instead, a new application-evaluation logic should be constructed around safety, ground-contact stability, operational accuracy, operator protection, and energy-consumption constraints.

### 4.1. Special Requirements of Agricultural Machinery Suspension

Chassis excitation in agricultural machinery is characterized by strong randomness, broad bandwidth, and strong time variation. Field ground is not a rigid and flat road surface. Its support stiffness is affected by soil moisture content, compaction degree, crop residues, ruts, and slope, showing significant nonlinearity and spatial nonuniformity. Tires or tracks on soft soil may also generate sinkage, slip, and redistribution of contact pressure, so the suspension system is subjected not only to road geometric unevenness, but also to changes in soil mechanical properties. For tracked chassis, track engagement, polygonal effects, and tension fluctuations can also induce periodic high-frequency excitation, further amplifying chassis vibration and structural fatigue risk [105,106].

Under off-road conditions, conventional rule-based algorithms such as Skyhook control often struggle to adapt adequately to abrupt impacts and time-varying loads. Actuator response delay caused by rough terrain can weaken feedback-control effectiveness, forcing the system to rebalance bump isolation, ground-contact stability, and handling safety [107]. According to chassis response characteristics, the dynamic challenges in off-road vehicles and agricultural machinery can be summarized into three categories. The first is lateral handling instability. In small or micro agricultural tractors without axle suspension, ground bumps may reduce the vertical force of the front wheels, thereby weakening the static friction of the steering wheels and inducing steering instability. This is also an important dynamic cause of rollover accidents in small agricultural machines on hilly and rough farm roads [108]. The second is loss of ground contact and re-impact. When a vehicle momentarily leaves the ground while crossing extreme obstacles or potholes and then contacts the ground again, the chassis may produce strong nonlinear or even chaotic vibration. For such abrupt responses, fractional delayed-feedback control can stabilize a chaotic response into periodic motion under the passivity constraints of semi-active control [109]. The third is abrupt load change. Mining vehicles, large agricultural trailers, and harvesting equipment operate under unloaded, partially loaded, and fully loaded conditions. A single set of passive suspension parameters cannot perform optimally across all three conditions. Adaptive variable-damping hydro-pneumatic suspension can shorten body stabilization time under full-load conditions through dynamic matching between the spool and piston-rod stroke [110]. In extreme off-road scenarios such as all-terrain vehicles, robust H∞ controllers based on Lyapunov–Krasovskii theory can also be used to suppress body pitch and roll responses [111].

In addition to chassis safety, complex excitations directly impair the operational accuracy of agricultural machinery. For example, surface residues, ridges and furrows, and implement excitations can reduce the stability of no-tillage seeding, straw returning, and precision seeding processes [112,113]. Chassis vibration can disturb the airflow field and seed-pickup qualification rate of precision seeders [114,115]. Vibration of headers, conveying troughs, and screening mechanisms may also cause crop loss and mechanical fatigue. Existing studies have shown that structural vibration-reduction optimization can reduce header shatter loss in rapeseed combine harvesting [116]. Therefore, the value of semi-active suspension in agricultural machinery lies not only in improving ride comfort, but also in helping improve operational accuracy and overall machine safety by suppressing coupled chassis–implement vibration.

To facilitate understanding of the agricultural engineering value of semi-active suspension from the perspective of application scenarios, Table 5 summarizes the dynamic challenges, semi-active control or related hardware schemes, and expected improvements for typical special agricultural machinery scenarios.

As shown in Table 5, the evidence base spans numerical models, experimentally calibrated simulations, all-terrain vehicle tests, damper benches, and agricultural field studies. The reported percentages are not directly rankable because vehicle mass, excitation spectra, speed, load, controller baseline, and performance metrics differ. Nevertheless, the comparison reveals a consistent maturity gap: agricultural seat and tracked-vehicle studies often remain at the simulation or component-validation level, whereas road-vehicle MR and preview-MPC studies have progressed further toward vehicle-level testing. Future agricultural research should, therefore, report both performance gains and the complete validation context.

### 4.2. Seat/Cab Suspension and Operator Protection

Agricultural machinery operators are usually exposed for long periods to low-frequency, large-amplitude, and multi-directional vibration. Vibration energy is transmitted to the human body through the seat, floor, and control mechanisms, potentially increasing lumbar spine injury and occupational health risks. Real-vehicle vibration tests on tracked combine harvesters have shown that under no-load idle, road driving, field driving, and simulated harvesting conditions, the vertical vibration of the operator seat exhibits obvious low-frequency characteristics, and the original seat can amplify vibration in some frequency bands. After improving the seat damping structure, the vibration acceleration at the seat support surface was reduced. These measurements show that seat/cab vibration isolation remains an important entry point for reducing WBV exposure in agricultural machinery [3,119]. Therefore, seat suspension is not only a comfort component, but also an important part of safety and human-factors engineering design in agricultural machinery.

Magnetorheological seat suspension has attracted attention because of its fast response, relatively compact structure, and wide damping-adjustment range. Within limited installation space, an MR seat suspension system based on linear active disturbance rejection control (LADRC) can reduce peak vertical acceleration of the operator under typical farm-road excitation, showing good low-frequency vibration-isolation capability [53]. However, the difficulty in seat-suspension design lies in the limited available dynamic travel. When low-frequency large-amplitude impacts exceed the operating range of the seat suspension, simply increasing damping may enhance impact transmission. Therefore, semi-active seat control must simultaneously consider human comfort, suspension dynamic travel, and end-stop impact risk. For example, LADRC simulations based on experimentally identified MR-damper characteristics reduced vertical seat-acceleration RMS by 15.0% relative to PID and 24.2% relative to passive suspension at 1 m/s on an ISO D-class road; the system-level result remains numerical rather than field-validated [53]. For the ANFIS-ADRC study, the values in its Table 5 (1.90 m/s^2^ for passive, 0.90 m/s^2^ for PID, and 0.59 m/s^2^ for ADRC) imply RMS reductions of 68.9% relative to passive and 34.4% relative to PID under the reported ISO C-class simulation. This table-derived interpretation is used because the ordering of the two percentages in the article prose is internally inconsistent; the training burden and lack of system-level hardware validation also constrain maturity [52].

**Figure 6:**
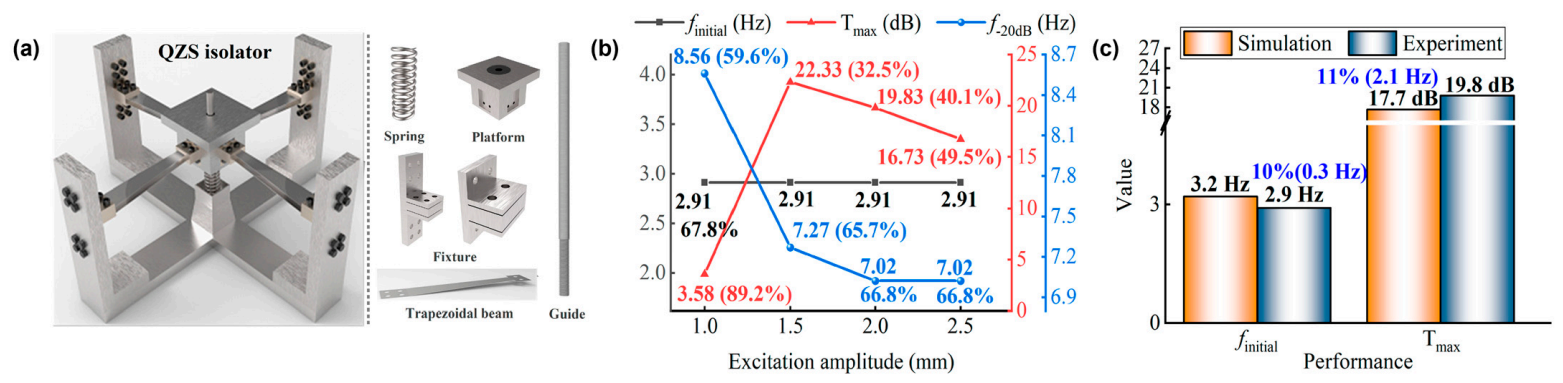
Structural configuration and low-frequency vibration-isolation performance of a trapezoidal-beam quasi-zero-stiffness (QZS) isolator: (**a**) prototype and principal components; (**b**) variations in initial isolation frequency, peak transmissibility, and the frequency corresponding to −20 dB transmissibility under different excitation amplitudes; and (**c**) comparison of finite-element predictions with experimental results. The results illustrate how the QZS mechanism broadens the low-frequency isolation range while maintaining agreement between numerical and experimental responses.

Quasi-zero-stiffness (QZS) isolators provide a structural route for extending the low-frequency vibration-isolation range without sacrificing static load capacity. As illustrated in Figure 6, the inclined trapezoidal beams buckle under an appropriate preload and provide nonlinear negative stiffness, while the parallel linear spring provides positive stiffness and supports the static load. The compensation between the positive and negative stiffness components forms a near-zero dynamic-stiffness region around the operating equilibrium [13]. Dynamic experiments showed that the proposed QZS isolator had a substantially lower initial isolation frequency than the equivalent linear isolator, while the comparison between finite-element simulation and experimental results supported the validity of the predicted low-frequency response. To further suppress resonance amplification and improve adaptability under large-amplitude impacts, adjustable MR damping can be integrated with nonlinear QZS structures, forming an MR-damping-based QZS isolator with controllable energy dissipation [19].

Figure 6 indicates that effective low-frequency isolation cannot be achieved simply by increasing damping. Instead, the equivalent dynamic stiffness around the operating equilibrium must be reduced while adequate static load capacity is preserved. In the cited trapezoidal-beam QZS isolator, the experimentally measured initial isolation frequency was substantially lower than that of the equivalent linear isolator, and the simulation–experiment errors in initial isolation frequency and peak transmissibility were approximately 10% and 11%, respectively [13]. However, the QZS region is sensitive to preload, payload variation, structural alignment, friction, and available displacement travel. MR damping can complement nonlinear stiffness compensation by suppressing resonance and limiting excessive displacement, but this introduces additional actuator, power-supply, control, and durability requirements [19].

It should be noted that although seat and cab suspension are directly meaningful for operator protection, they cannot fully replace chassis suspension. If chassis vibration has already reduced tire/track ground contact, destabilized implement attitude, or increased whole-vehicle rollover risk, improving seat vibration isolation alone cannot fully address overall machine safety and operational-performance issues. Therefore, local vibration isolation should be regarded as one level within the semi-active suspension system of agricultural machinery, rather than the whole objective.

### 4.3. Tractors, Tracked Chassis, and Rollover Prevention Control

Tractors, tracked vehicles, and hilly-operation platforms are the most representative chassis objects in semi-active suspension applications for agricultural machinery. Compared with passenger cars, this type of equipment has a higher center of gravity, larger load variation, uneven operating environments, low speed but large impact amplitudes, and other distinctive characteristics. Their suspension-control objectives include not only ride comfort, but also sustained tire/track ground contact, slope attitude stability, load-transfer control, and rollover-risk suppression.

**Figure 7:**
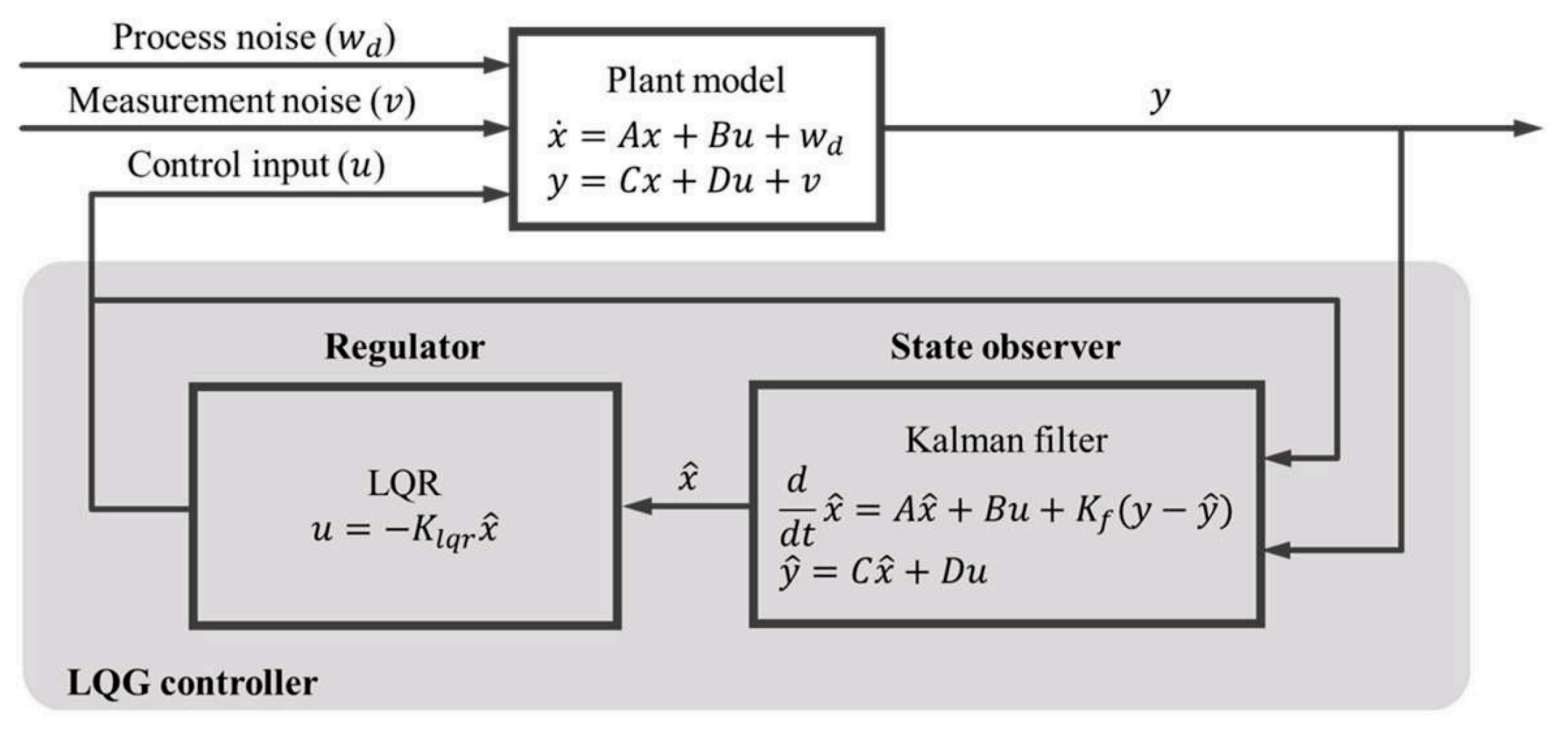
Linear quadratic Gaussian (LQG) control architecture for an agricultural-tractor semi-active cabin suspension. A Kalman-filter-based observer reconstructs the unmeasured cabin states from limited rear-suspension feedback, while the LQR controller determines the target force of the rear hydro-pneumatic suspension, illustrating a low-sensor-count route to optimal vibration control.

Agricultural tractors require suspension controllers specifically formulated for their structural characteristics rather than directly transferred from passenger-car models. In a representative study, Ahn et al. developed a linear four-degree-of-freedom half-car model for a tractor cabin with an asymmetric suspension configuration, consisting of a passive rubber mount at the front and a hydro-pneumatic semi-active suspension at the rear. The controller combined linear quadratic regulator (LQR) optimal control with a Kalman-filter-based state observer, forming an LQG semi-active control framework. Considering the limited sensing resources available on agricultural machinery, rear-suspension deflection was used as the directly measured feedback variable, rear-suspension velocity was obtained by differentiation, and the remaining states required by the full-state controller were estimated by the observer [120]. The corresponding control structure is shown in Figure 7.

Under ISO 8608 Class A–C road excitations at a tractor speed of 5 km/h, the observer achieved reported accuracies of approximately 83% and 79% for cabin vertical velocity and acceleration, respectively. Compared with the passive rear rubber-mount configuration, the LQG-controlled semi-active suspension reduced the peak cabin vertical acceleration by 39.78–48.97% and the corresponding RMS value by 41.12–47.06% across the three road classes [120]. A further parametric study over operating speeds of 3–10 km/h reported peak- and RMS-acceleration reductions of 35.64–57.34% and 30.98–53.95%, respectively. These results demonstrate the potential of observer-based optimal control under limited sensing conditions. However, the evidence was obtained through numerical simulation using actual tractor parameters and identified suspension characteristics; no real-time ECU implementation, hardware-in-the-loop testing, vehicle-level testing, or agricultural field validation was reported. Consequently, this study should be regarded as a simulation-validated, low-sensor-count control architecture rather than a field-ready suspension system.

For tracked vehicles, the suspension assessment should include track-tension dispersion and engagement/polygon excitation in addition to body acceleration. A 43 t tracked-vehicle model reported an approximately 15% reduction in track-tension standard deviation and a 23–37% improvement in vertical vibration attenuation at 50 km/h on a grade-E road under MR-Skyhook control; the evidence is numerical and should not be generalized directly to agricultural field speed or soil conditions [106]. This case nevertheless shows why track-contact metrics and high-frequency engagement dynamics must enter agricultural suspension evaluation.

In tracked chassis systems, track engagement and polygonal effects induce periodic high-frequency vibration, especially in triangular rubber tracks, chassis without independent guide wheels, or lightweight tracked chassis. High-frequency chatter not only affects driving comfort but may also change track tension and ground-pressure distribution, thereby aggravating soft-soil compaction or reducing adhesion stability. Independent Skyhook MR semi-active suspension, differential-viscosity magnetorheological fluids, and coordinated control of track-tensioning systems provide feasible ideas for suppressing tracked-chassis impacts and optimizing ground-pressure distribution [106,121,122]. These studies indicate that suspension control for tracked agricultural machinery should not focus only on body acceleration, but should also consider track–soil contact, tension fluctuation, and soil-compaction risk.

In hilly and mountainous regions, omnidirectional chassis attitude leveling is a prerequisite for operational safety and accuracy [123]. During slope operation, vehicle roll angle, load transfer ratio, and steering-wheel ground-contact state directly determine rollover risk and handling stability. To address slope-induced attitude instability, sliding-mode synchronous control based on a three-layer-frame hydraulically interconnected structure can achieve attitude coordination under terrain disturbances [124]. A series structure consisting of a servo electric cylinder, spring, and damper combined with fuzzy PID control can also be used for hilly chassis leveling and impact buffering [125,126]. These schemes reflect the trend that agricultural chassis suspension is expanding from single-function vibration reduction toward attitude leveling and safety control.

Extreme rollover prevention is an important safety objective in chassis control for agricultural machinery with a high center of gravity. Integrating an ISD network into pneumatic damping systems can suppress dynamic-travel amplification under large sideslip conditions [127]. A 4/3 directional control valve (DCV)-based roll-interconnected semi-active suspension (RISAS) can use suspension compression potential energy to redistribute fluid and improve the load transfer ratio (LTR) under passive conditions [128]. Therefore, semi-active suspension design for hilly and sloping agricultural machinery should not focus only on vertical vibration suppression, but should also cooperate with hydraulic leveling, steering control, and chassis attitude control to jointly improve ground-contact stability and safety margins under critical rollover conditions.

### 4.4. Precision Implements and Operational Accuracy Assurance

Another important target affected by agricultural machinery vibration is precision-implement performance. For modern intelligent agricultural equipment, operational performance depends strongly on the relative stability between the chassis and implements. Chassis vibration, pitch, and roll are transmitted through the frame, boom, header, and seeding units to the implement end, causing spraying deviation, nonuniform seeding, increased header loss, and variable-rate application errors. Therefore, semi-active suspension in agricultural machinery should support not only ride comfort but also operational accuracy.

Wide-boom sprayers are typical vibration-amplification scenarios. Because booms or cantilever structures are large and highly flexible, small chassis roll or rolling motion can be amplified by the boom and cause height error at the boom tip and nonuniform spray coverage. To address this problem, introducing feedforward PID, fuzzy PID, or semi-active leveling control into trapezoidal suspension or double-wishbone suspension can isolate chassis roll oscillation and control spatial errors at the boom end [117,129,130,131]. These studies indicate that key evaluation indices for sprayer chassis control are not only body acceleration, but also boom height, lateral attitude, and target-spraying trajectory stability.

Precision seeding, variable-rate fertilization, and intelligent weeding are also affected by chassis vibration. Surface residues, ridges, furrows, and broadband frame vibration change the ground-contact pressure, airflow field, and seed adsorption state of seeding units, thereby reducing seeding qualification rate and operational consistency [114,115]. Under high-speed operation, frame structural modes, suspension dynamic travel, and operating speed are coupled. If key resonance bands cannot be avoided, chassis vibration may be further amplified at the implement end. Methods based on time-frequency-domain signal extraction, modal avoidance, and active/semi-active reduced-order control can be used to identify key vibration bands and provide low-dimensional state inputs for controllers [118].

Vibration problems in harvesting and straw-processing equipment are even more complex. Headers, conveying troughs, vibrating screens, and chopping mechanisms themselves generate periodic excitations. Chassis vibration can superimpose with these internal excitations, forming multi-source coupled vibration. For combine harvesters, balers, and straw-returning equipment, vibration affects not only operator comfort, but also crop conveying, screening efficiency, stubble-crushing quality, and frame fatigue life [112,113]. Therefore, combining semi-active suspension with implement structural damping optimization, transmission-path analysis, and operation-speed control is an important direction for improving the stability of high-speed precision operations.

### 4.5. Application-Oriented Design Priorities

In summary, the design objectives of semi-active suspension for agricultural machinery and off-road vehicles are fundamentally different from those for on-road passenger cars. Passenger-car suspension is mainly designed around a trade-off between comfort and handling stability, whereas agricultural machinery must also simultaneously face soil time variation, abrupt load changes, operating-implement disturbances, slope rollover risk, and long-term environmental degradation. Therefore, using body vertical acceleration RMS as the sole objective can easily overlook more critical agricultural engineering requirements such as chassis ground contact, operational accuracy, and safety boundaries.

This review argues that the design priorities of semi-active suspension for special agricultural machinery and complex off-road equipment should be reconstructed as follows: safety, including rollover prevention and structural protection; ground-contact stability, including tire/track adhesion and traction; operational accuracy, including spraying, seeding, weeding, and harvesting; operator comfort; and energy consumption. This ranking does not imply that comfort is unimportant. Rather, it emphasizes that agricultural semi-active suspension should be designed as a system-level control technology that coordinates actuator, chassis, soil, implement, and operator dynamics.

Section summary: Off-road and agricultural machinery impose requirements that differ from those of passenger cars. The core value of semi-active suspension is not simply reducing body acceleration, but coordinating chassis safety, ground-contact stability, operational accuracy, and human–machine protection.

## 5. Validation, Engineering Deployment, and Future Challenges

The preceding sections reviewed semi-active suspension from the perspectives of actuators, modeling, control algorithms, and agricultural applications. However, engineering deployment depends not only on simulation-level vibration-reduction performance, but also on whether evaluation indices, validation procedures, sensing and computing platforms, actuator energy consumption, and long-term reliability meet agricultural operating requirements. Therefore, this section discusses multi-objective evaluation, validation hierarchy, real-time deployment, reliability, energy constraints, and future research directions.

### 5.1. Multi-Objective Evaluation Indices and Validation Hierarchy

The evaluation system of semi-active suspension is shifting from a single ride-comfort index toward multi-objective comprehensive evaluation. Traditional vehicle suspension research usually uses root mean square (RMS) body vertical acceleration, suspension dynamic travel, and dynamic tire load as core indices. These indices reflect the basic trade-off among ride comfort, suspension working space, and handling stability. However, for agricultural machinery, these indices alone are insufficient to evaluate the engineering value of a control system. Agricultural machinery also needs to consider whole-body vibration exposure, slope rollover risk, track ground pressure, implement-end attitude, trajectory tracking error, operational accuracy, and energy consumption.

**Figure 8:**
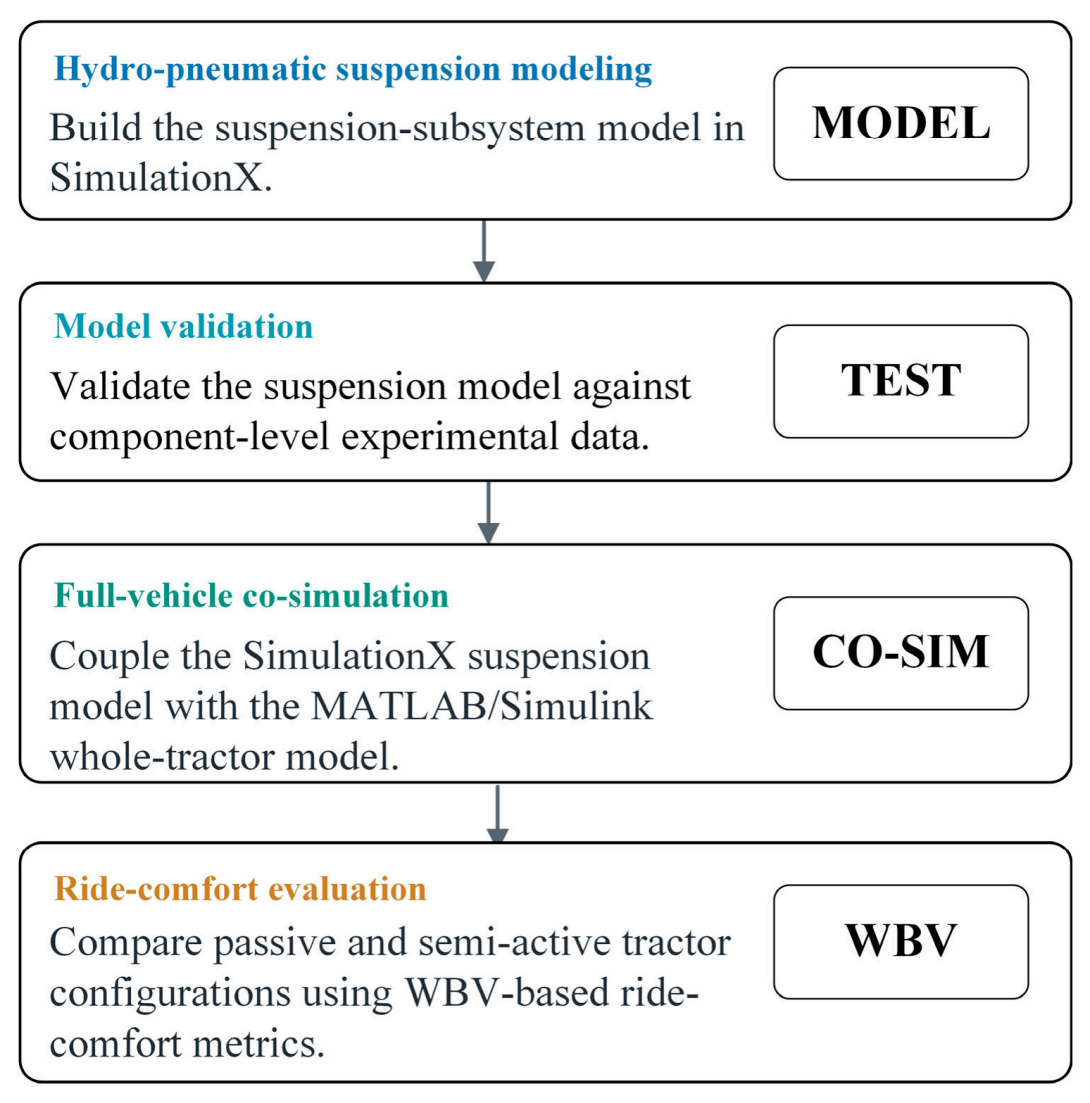
Modeling and validation workflow for a hydro-pneumatic semi-active cab suspension of an agricultural tractor. The workflow links SimulationX suspension-subsystem modeling and component-level experimental validation with MATLAB/Simulink whole-tractor co-simulation and WBV-based comparison of passive and semi-active configurations, thereby connecting model credibility with whole-vehicle ride-comfort assessment.

For agricultural tractors, engineering evaluation of semi-active suspension should not focus only on body acceleration or suspension dynamic travel, but should also jointly consider suspension structural modeling, control algorithms, model validation, and operator comfort indices. Sim et al. developed a SimulationX-based model of a hydro-pneumatic semi-active cab suspension for an agricultural tractor and coupled it with a MATLAB/Simulink whole-vehicle model. The suspension model was validated using experimental data, and the passive and semi-active configurations were compared using WBV-related ride-comfort indices [132]. Based on this representative study, Figure 8 synthesizes a progressive workflow from suspension-subsystem modeling and component-level experimental validation to whole-vehicle co-simulation and WBV-based ride-comfort assessment.

Therefore, evaluation of semi-active suspension in agricultural machinery should cover four dimensions: operator comfort, chassis safety and ground-contact stability, operational accuracy, and engineering deployment. These dimensions should be validated progressively through numerical simulation, high-fidelity simulation, bench/HiL validation, and agricultural field validation. Table 6 summarizes the multi-objective evaluation indices, functions, limitations, and representative references for agricultural semi-active suspension systems, whereas Table 7 presents the corresponding validation hierarchy from simulation to field testing.

In addition to evaluation indices, engineering deployment also requires a progressive validation hierarchy, as summarized in Table 7.

As shown in Table 7, the transition of semi-active suspension from algorithm research to agricultural engineering application requires a progressive validation process comprising numerical simulation, high-fidelity simulation, bench/HiL validation, and agricultural field validation. Different levels serve control-law screening, structural boundary identification, hardware real-time validation, and real-condition evaluation, respectively, helping avoid overstatement of algorithm effectiveness based solely on simulation results. For agricultural machinery, final evaluation should not focus only on body acceleration and suspension dynamic travel, but should also include engineering indices such as ground-contact stability, operational accuracy, rollover-prevention capability, energy consumption, and long-term reliability.

In chassis–implement collaborative control, suspension is no longer limited to vertical vibration reduction, but needs to collaborate with trajectory tracking, traction control, and operating-implement attitude maintenance. Under limit handling conditions, implicit nonlinear model predictive control (NMPC) can combine vibration reduction with active roll intervention to track target yaw rate and improve chassis attitude response [137]. For agricultural machinery, the focus of collaborative control is not the limit handling of high-performance vehicles, but the integrated coordination of chassis, implement, and traction. For example, when intelligent tractors are coupled with rotary tillers, seeders, or balers, suspension needs to compensate through feedforward or feedback for time-frequency-domain vibration induced by implement lifting, cutting, and ground excitation, thereby balancing trajectory accuracy and operation smoothness [46,97]. In electric-drive or hybrid agricultural machinery, road-preview robust MPC is also coupled with motor torque distribution and slip-ratio control. Suspension control should suppress body vibration while avoiding disruption of traction output and sideslip-prevention control [138,139].

### 5.2. Real-Time Implementation: Perception, Computation, and Execution

For semi-active suspension control to be implemented in agricultural machinery, three real-time implementation problems must be solved: perception, computation, and execution. The first is perception. In off-road and farmland environments, mud and water, dust, crop occlusion, strong light, weak light, vibration shocks, and electromagnetic interference all affect the reliability of vision, LiDAR, IMU, wheel-speed, pressure, and displacement sensors. In addition to road and body states, agricultural machinery suspension control must also focus on operation loads and soil–implement interaction information. For example, the tillage resistance of a tractor-mounted plough can be estimated through force signals at upper-link and lower-link hinge points and a spatial vector mechanics model. Such load information can serve as an important input for future chassis–implement collaborative control, traction-suspension joint control, and soil-adaptive regulation [140,141,142,143]. Although terrain preview can improve feedforward control capability, its stability remains uncertain at night, after rain on muddy ground, under crop cover, and in heavy dust. Future preview systems should fuse lightweight vision, virtual reference profiles, IMU-based state estimation, and forward-looking elevation sensing. Such multi-source fusion can improve robustness under complex field conditions [89,90,140,141,142,144].

Real-time applicability should be evaluated over the complete sensing–computation–execution chain rather than by controller computation alone. The end-to-end delay comprises terrain-perception latency, state-estimation time, controller execution, communication, electromagnetic or hydraulic actuator response, and mechanical force build-up. A strategy is practically real-time only when the worst-case end-to-end delay remains below the allowable control period under rough-terrain impacts and time-varying loads. Accordingly, future studies should report processor type, sampling frequency, worst-case execution time, communication protocol, actuator response, packet loss, and fail-safe fallback. The approximately 10 ms MR-damper hardware response and the approximately 60 ms VIO perception time refer to different stages of the control chain. They should neither be added directly nor interpreted as universal closed-loop delays [8,104].

Agricultural sensing also requires explicit uncertainty and fallback management. Full-text field evidence shows that dense foliage degrades GNSS, extreme lighting remains insufficiently tested for camera-based VIO, and sparse features can weaken vision-based localization. A recent agricultural-robotics review further identifies camera sensitivity to lighting and occlusion, inertial drift, and the complementary roles of camera, LiDAR, GNSS, and IMU fusion; it also recommends fail-safe stopping or human notification when uncertainty cannot be resolved [145]. An agriculture-specific LiDAR review documents field-boundary, ridge, obstacle, and crop-recognition uses, while emphasizing sensor selection, mounting, testing, and accuracy rather than treating LiDAR as an intrinsically reliable preview source [146]. Preview-suspension studies likewise identify profile-estimation error, measurement noise, communication delay, and fusion accuracy as performance-limiting factors [6,104]. Edge-computing architectures can reduce network dependence and support local multi-sensor fusion, but field communication reliability, computing resources, and energy remain constraints rather than evidence of a validated suspension controller [147]. Agricultural field systems also use inclination and displacement-related signals for boom-posture and hydraulic-cylinder control; however, the reviewed studies primarily validate measurement and control performance rather than dedicated sensor-fault detection or fault-tolerant operation [117].

Table 8 summarizes the principal sensing and communication failure modes, their control consequences, and the corresponding degraded-operation strategies.

The second is computation. Advanced algorithms such as MPC, NMPC, DRL, and PPO-NODE usually have high computational complexity, whereas mass-produced agricultural machinery controllers are strictly limited by cost, power consumption, memory capacity, and real-time requirements. Direct deployment of high-dimensional receding-horizon optimization or deep networks on low-cost ECUs may result in excessive computation time, deadline misses, or reduced control effectiveness under high-frequency impact [2].

Therefore, lightweight control algorithms are critical for deploying semi-active suspension in agricultural machinery. The reviewed routes include reduced-order or invertible models, explicit MPC, offline-trained and compressed learning-based control, and cloud–edge collaboration. However, their evidence levels are not equivalent. Reduced-order models and explicit MPC have direct suspension-related modeling or vehicle-deployment evidence [21,23,59], whereas network pruning, quantization, knowledge distillation, event-triggered updating, and cloud–edge suspension control remain partly prospective in the reviewed agricultural suspension literature [2,60,147]. To distinguish directly reported implementation evidence from engineering interpretation, Table 9 classifies these routes according to their reported basis, online computational burden, communication dependence, and deployment or fail-safe considerations.

The classification in Table 9 shows that lightweight deployment cannot be evaluated from nominal algorithm complexity alone. Processor-specific execution time, memory use, energy consumption, communication reliability, deadline-miss behavior, and fallback performance should be reported together. Explicit MPC has direct vehicle-level deployment evidence among the listed routes [59], whereas compressed learning-based and cloud–edge approaches still require processor-in-the-loop, HiL, vehicle, and agricultural field validation [2,60,147]. Cloud-dependent perception, optimization, or model-updating functions should remain subordinate to a locally executable safe damping controller.

The third is execution. The actual output of semi-active suspension actuators is often affected by power-drive circuits, electromagnetic coils, proportional valves, hydraulic hysteresis, and mechanical friction. Even if the control algorithm calculates an ideal damping command, the actual damping force may still deviate from the target value if the drive-circuit response is slow, coil-current tracking error is large, or proportional valves have dead zones. Therefore, real-time control systems need to include actuator delay and drive nonlinearity in the control logic. For MR dampers, attention should be paid to coil-current build-up time, thermal attenuation, and force lag. For CDC dampers, proportional-valve actuation frequency, spool wear, and hydraulic hysteresis must be considered. For self-powered or energy-regenerative dampers, the reverse influence of the energy-harvesting circuit on equivalent damping also needs to be handled.

Broadband high-intensity noise in agricultural machinery also increases the difficulty of signal conditioning. Time-frequency and modal-decomposition methods, including EEMD, CEEMDAN, and PAVMD, can extract load spectra, weak target signals, and dominant vibration bands. These outputs can support state estimation, bench calibration, and fatigue analysis [118,148,149,150,151]. However, some methods involve long iterative processes and are more suitable as offline analysis or edge-side preprocessing tools. In real-time control, low-delay, interpretable, and easily deployable signal-processing schemes should still be prioritized. Such signals can also be used to construct RMS prediction models or directly drive online optimization control [152,153].

In summary, real-time deployment of semi-active suspension is not a single-algorithm problem, but a closed-loop perception–computation–execution problem. The perception side must balance robustness in complex environments and cost. The computation side must balance control performance and low delay. The execution side must balance response speed, power consumption, and service life. Only an integrated perception–computation–execution chain can move semi-active suspension from bench validation to vehicle-level testing and agricultural field deployment.

### 5.3. Reliability, Energy Consumption, and Self-Powered Systems

Agricultural machinery operates for long periods in mud and water, dust, temperature differences, high humidity, and high-load impact environments. Reliability of semi-active actuators is, therefore, a key factor affecting engineering application. MR dampers may face fluid sedimentation, seal wear, coil heating, thermal attenuation, and force lag. CDC dampers may be affected by proportional-valve wear, oil contamination, and spool hysteresis. Self-powered dampers must simultaneously ensure the reliability of mechanical vibration reduction, power generation units, energy management, and control circuits. For agricultural machinery, actuators must perform well not only in short-term bench tests, but also maintain stable performance under long-term load cycling, mud and water erosion, and limited maintenance conditions.

Chassis electrification further strengthens energy-consumption constraints on suspension systems. Pure electric orchard tractors, greenhouse tracked electric tractors, hybrid agricultural machinery, and electric-drive chassis need to allocate limited energy among driving, steering, control, and operating implements. This requires suspension systems to reduce control power consumption as much as possible and avoid frequent high-energy actuation [139,154,155,156,157]. In addition, electromagnetic disturbances in electric-drive systems may couple with road excitation, so suspension control needs to be considered collaboratively with the whole-vehicle power domain and energy management [158,159].

Self-powered or energy-regenerative semi-active suspension provides new ideas for low-power agricultural equipment, but its core contradiction lies in the performance trade-off between energy harvesting and vibration isolation. Enhanced energy recovery may change equivalent damping characteristics and cause damping jumps or vibration amplification under some high-frequency conditions. Therefore, self-powered suspension should not be evaluated only by energy recovery efficiency, but should comprehensively consider vibration-isolation performance, power stability, circuit reliability, and control safety boundaries [134,160,161,162]. For agricultural machinery, a more realistic route may be low-power semi-active control plus local energy recovery plus self-sensing assistance: vibration reduction and safety should first be prioritized, and recovered energy can then support sensing, low-power control, and state monitoring.

### 5.4. Future Technical Roadmap

Based on the preceding analysis, future work should be organized as testable engineering programs rather than broad algorithmic aspirations. Each priority below is specified through its current gap, technical bottleneck, near-term task, evaluation metric, and validation route.

First, harsh-environment actuator health and fault tolerance. The present gap is the lack of standardized degradation data linking seal wear, MR-fluid sedimentation, temperature, coil heating, valve hysteresis, and force attenuation to controller performance. The bottleneck is that short bench tests do not represent combined mud, dust, thermal cycling, and shock loads. Priority tasks are accelerated multi-stress testing, health-indicator extraction, probabilistic parameter updating, remaining-useful-life estimation, and passive-safe degraded control. Metrics should include force-tracking error, leakage, temperature rise, attenuation rate, fault-detection delay, and safe-fallback success. Validation should progress from component endurance rigs to HiL and seasonal field trials [7,20].

Second, confidence-aware terrain and soil preview. Existing preview systems mainly estimate road geometry, whereas agricultural control also requires soil stiffness, moisture, sinkage, slip, track pressure, and implement-load change. The bottlenecks are heterogeneous sensing, time synchronization, contamination, and uncertain prediction under sparse features. Priority tasks include fusion of camera, LiDAR, IMU, and wheel/track signals, confidence propagation, soil-state estimation, and graceful degradation when preview fails. Metrics should report terrain-prediction error, end-to-end latency, confidence calibration, fault-recovery time, vibration reduction, and ground-contact improvement. Validation should use public farm-road/soil datasets followed by orchard, slope, soft-soil, and tracked-vehicle field tests [104,145,146].

Third, safe and lightweight intelligent control. The main gap is that MPC and DRL studies rarely report worst-case execution time, memory, processor utilization, training coverage, or certified safety behavior on low-cost agricultural ECUs. The bottleneck is the simultaneous need for nonlinear adaptation, explicit constraints, interpretability, and bounded latency. Priority tasks include reduced-order state models, explicit MPC, network pruning/quantization, knowledge distillation, offline training and online inference, event-triggered updates, and safety layers for mechanism–data fusion control. Metrics should include execution-time distribution, memory and energy use, constraint violations, robustness to load/soil shifts, and fail-safe transitions. Validation should proceed through processor-in-the-loop, HiL, vehicle-level, and field testing rather than simulation alone [59,147].

Fourth, digital twins, predictive maintenance, and energy coordination. Agricultural suspension currently lacks a continuously updated virtual representation connecting actuator health, structural dynamics, operating loads, and energy use. The bottlenecks are model drift, uncertain parameters, intermittent communication, and cross-machine generalization. A combine harvester digital-twin field case demonstrates that lightweight virtual-physical mapping and online prediction are feasible for agricultural equipment, although it did not address suspension directly [163]. Priority tasks are suspension-specific state synchronization, uncertainty-aware parameter updating, health prediction, maintenance scheduling, and integration with whole-vehicle energy management. Metrics should include prediction error, update latency, health-state accuracy, maintenance lead time, and net energy benefit.

Fifth, standardized multi-objective evaluation and open field validation. The gap is the absence of common farmland excitation spectra, open datasets, benchmark controllers, durability cycles, and reporting rules. Priority tasks are to define repeatable scenarios for farm roads, standard tracks, slopes, soft soil, tracked engagement, implement excitation, and load transitions, while establishing repeatable WBV measurement and reporting procedures suitable for agricultural machinery. Every study should report machine configuration, excitation, speed, load, sampling, controller hardware, delay chain, actuator limits, comfort, ground contact, safety, operational accuracy, energy, reliability, and statistical uncertainty. A staged benchmark should require numerical, bench/HiL, vehicle, and multi-season field evidence before commercialization claims.

## 6. Conclusions

Available evidence indicates that several semi-active suspension components and road-vehicle implementations have reached relatively advanced validation stages. However, maturity remains uneven across actuator types, control strategies, and operating scenarios, while agricultural field evidence remains limited. The central scientific gap is not the absence of control algorithms, but the lack of a traceable engineering chain connecting harsh-environment actuator capability, uncertainty-aware reduced-order models, end-to-end real-time control, multi-objective agricultural metrics, and repeatable field validation.

Among the reviewed options, MR and CDC dampers have existing component- and vehicle-level evidence supporting consideration for near-term deployment, although agricultural endurance, maintenance-interval data, and multi-season field evidence remain limited. High-fidelity MBD, FEM, and DEM–FMBD models should identify modes, vibration paths, contact conditions, and safety boundaries offline, whereas online controllers should use validated reduced-order or explicitly constrained models. Classical robust and adaptive methods retain advantages on low-cost ECUs; explicit MPC is attractive when constraints and preview information are available; and mechanism–data fusion control is promising when explicit safety layers, processor-specific timing, and hardware validation accompany learning-based modules.

For agricultural machinery, performance must be judged through operator WBV, suspension travel, tire/track contact, rollover risk, implement accuracy, energy use, reliability, and validation maturity. The highest priorities are, therefore, (i) multi-stress actuator durability and health monitoring, (ii) confidence-aware terrain and soil preview with degraded operation, (iii) lightweight constraint-aware control with reported worst-case timing, and (iv) standardized HiL, vehicle, and field benchmarks supported by repeatable vibration measurement and reporting procedures. Accordingly, future evaluation should consider not only simulation-level vibration reduction, but also transparent actuator endurance, processor-specific real-time performance, fault-tolerant sensing, implement-level operational performance, and repeatable multi-season field validation. Progress on these priorities will determine whether semi-active suspension moves from promising isolated demonstrations to maintainable and certifiable intelligent agricultural systems.

## Figures and Tables

**Table 1:** Comparison of representative recent reviews on suspension systems and the present review.

Review	Primary Scope	Hardware Scope	Control Focus	Agricultural Coverage	Main Emphasis	Key Distinction
Kimball et al. (2024) [2]	Road vehicles	Active/semi-active; active dominant	Adaptive and RL control	Limited	Learning-control taxonomy	Comprehensive taxonomy; limited agricultural deployment and hardware validation
Theunissen et al. (2021) [6]	Road vehicles	Active and semi-active	Preview, LQ/H∞, MPC	Not covered	Preview horizon and actuator bandwidth	No systematic treatment of soil interaction, farmland sensor failure, or implement coupling
Wang et al. (2024) [4]	Automotive MR suspension	MR dampers	Classical, robust, and intelligent control	No dedicated coverage	MR technology and applications	No systematic evaluation of field durability or agricultural applicability
Yu et al. (2024) [5]	Road vehicles	Production-oriented active systems	Active and cooperative control	Not covered	Industrial deployment and packaging	Not focused on semi-active/MR systems or agricultural machinery
Chen et al. (2025) [3]	Agricultural machinery	Semi-active seat suspension	Fuzzy, sliding-mode, and adaptive control	Seat-focused	WBV and seat isolation	Excludes chassis, tracked vehicles, and implements
This review	On-road–off-road–agricultural chain	MR, CDC, regenerative, and QZS systems	Robust, MPC, DRL, and fusion	Chassis, cab, tracked systems, and implements	Durability, deployment, and field validation	Integrates engineering evidence and identifies validation and reporting gaps

**Note:** MR, magnetorheological; CDC, continuous damping control; QZS, quasi-zero stiffness; RL, reinforcement learning; WBV, whole-body vibration; LQ, linear-quadratic; H∞, H-infinity; MPC, model predictive control; DRL, deep reinforcement learning.

**Table 2:** Evidence-traceable comparison of representative semi-active actuation and vibration-isolation technologies.

Technology	Performance	Power/ Energy	Durability/ Maintenance	Validation	Agricultural Interpretation	Ref.
Linear/ hybrid MR damper	~10 ms response for one hybrid-valve design	Power data: NR	O-ring wear test: 200–300 min; service life/maintenance interval: NR	Bench/ vehicle seal-wear tests; agricultural endurance: NR	Rapid impact-control potential; sealing, contamination, and thermal constraints	[7,8]
Rotary MR damper	Comfort improvement: ≤21%; velocity-response reduction: ≤52%; seat transmissibility reduction: >20%	Power data: NR	Service life, endurance cycles, and maintenance interval: NR	Prototype/ seat tests and simulation; field validation: NR	Compact for narrow spaces; farmland durability unverified	[9,10]
CDC damper	Simulated peak reductions: suspension deflection, 49.6%; body acceleration, 50%; dynamic load, 50%	Power data: NR	Service life, temperature-dependent attenuation, and maintenance interval: NR	Bench-informed model and vehicle simulation; field validation: NR	Potential hydraulic route; superiority over MR dampers not established	[11]
Regenerative/self-powered damper	Maximum damping force: 4156 N; adjustable range: 6.25	Peak recovered power: 29.1 W; recovery efficiency: 11.2–24.6%	Service life, endurance cycles, and maintenance interval: NR	Modeling, simulation, and prototype testing; long-term field validation: NR	Auxiliary sensing power; energy-recovery/ isolation trade-off	[12]
QZS/bio-inspired isolator	Experimental initial isolation frequency: 2.91 Hz	Continuous actuation power: N/A; cross-design data: NR	Structural endurance, environmental resistance, and maintenance interval: NR	FEA and static/ dynamic tests; vehicle/field validation: NR	Local low-frequency isolation; payload and travel constraints	[13]

**Note:** NR, not reported; N/A, not applicable. The “Performance,” “Power/energy,” “Durability/maintenance,” and “Validation” columns contain source-reported evidence, whereas the “Agricultural interpretation” column represents author synthesis. Test-exposure data are not treated as service-life or maintenance-interval evidence unless explicitly defined in the original study. Non-harmonized test conditions preclude direct cross-study ranking.

**Table 3:** Comparison of representative nonlinear damper models and uncertainty-oriented surrogate approaches for semi-active control.

Model	Identification	Representation Capability	Online Burden	Validation Evidence	Engineering Role and Limits	Ref.
Modified Bouc–Wen	Many parameters; offline optimization	Strong hysteresis representation	High burden; difficult inversion	Bench-calibrated; primarily used in offline simulation	High-fidelity reference; unsuitable for low-cost online inversion	[21,22]
Prandtl–Ishlinskii	Fewer parameters; structured identification	Hysteresis with analytical inverse	Low to moderate	Bench tests and real-time tracking demonstrations	Low-delay inverse control; drift compensation needed	[21]
Algebraic/magic formula	Few parameters; curve fitting	Nonlinear fitting within the calibrated range	Low	Bench-calibrated and embedded in controllers	Suitable for MPC/ECU; extrapolation risk	[15,23]
Hammerstein/neural network	Data-intensive	Dynamic lag and complex hysteresis	Moderate to high; model compression needed	Predominantly numerical and bench validation	Residual/inverse model; physical constraints required	[24,25]
Probabilistic/surrogate model	Bayesian calibration + surrogate	Parameter uncertainty and response propagation	High offline; low online	MR-damper bench data with numerical uncertainty analysis	Digital-twin updating; not a life-prediction model	[20]

**Table 4:** Evidence-traceable comparison of semi-active suspension control strategies.

Method	Model/Constraints	Reported Timing	Adaptation	Validation	Agricultural Interpretation	Ref.
Skyhook/Groundhook	Low model dependence; rule-based constraints	Processor timing/WCET: NR	Fixed-rule coverage	Bench and vehicle evidence	Practical baseline; limited adaptation to varying soil and loads	[1]
ADRC/LADRC	Low-to-moderate model reliance; constraints mainly indirect	Processor timing and WCET: NR	Observer-based rejection; tuning/noise sensitive	Bench and simulation evidence	Promising under variable loads; field timing and fail-safe evidence limited	[52,53]
SMC	Moderate model reliance; constraints mainly indirect	Processor timing and WCET: NR	Strong robustness; chattering dependent	Simulation and selected bench evidence	Useful for nonlinear uncertainty; actuator-wear effects need hardware validation	[54,55]
Fuzzy/adaptive	Low-to-moderate model reliance; limited explicit constraints	Processor timing and WCET: NR	Adaptation depends on rule and tuning coverage	Predominantly simulation and bench evidence	Implementation-friendly; transfer beyond calibrated rules uncertain	[11,56]
MPC/NMPC	High model reliance; explicit constraint handling	WCET: NR in most reviewed studies	Effective when the prediction model remains valid	Simulation with selected HiL/vehicle evidence	Multi-objective capability; solver burden and model mismatch constrain low-cost ECUs	[57,58]
Explicit MPC + preview	Moderate model reliance; explicit constraint handling	NVIDIA Jetson AGX Xavier deployment; WCET: NR	Effective within the calibrated model and preview range	Full-scale ATV road test	Seat-weighted RMS reduced by 29.6%; pitch/roll reduced by 24.1%/26.7%; field transfer: NR	[59]
DRL/PPO/DDPG	Low explicit model reliance; constraints require reward or safety-layer design	Processor timing and WCET: NR in most reviewed studies	Potentially adaptive but dependent on training coverage	Predominantly simulation evidence	Generalization, interpretability, certification, and field robustness insufficient	[2,60]
Mechanism–data fusion	Physical model with learned residual	~2.05 ms on i9-12900KF PC; ECU timing/WCET: NR	Mechanism-based residual adaptation	Numerical simulation; HiL and vehicle validation: NR	Interpretability potential; processor-specific field robustness unverified	[25]

**Note:** NR, not reported; WCET, worst-case execution time; HiL, hardware-in-the-loop. The “Reported timing” and “Validation” columns contain source-reported evidence, whereas the “Model/constraints,” “Adaptation,” and “Agricultural interpretation” columns represent author synthesis. Reported times are processor- and implementation-specific and are not WCET unless explicitly stated. Non-harmonized models, processors, excitations, and validation procedures preclude direct cross-method ranking.

**Table 5:** Evidence-based comparison of representative suspension and vibration-control applications relevant to agricultural machinery.

Application	Hardware/ Control	Target	Reported Outcome	Validation	Deployment Burden	Evidence Boundary	Ref.
Tracked vehicle (potential agricultural transfer)	Rotary MR damper with Skyhook	Track tension and chassis vibration	Track-tension SD reduced by ~15%; vertical vibration reduced by ~23–37%	High-fidelity numerical model	High force; high structural complexity	Numerical only; no agricultural-soil or field validation	[106]
ATV (transferable off-road evidence)	MR damper with preview-based explicit MPC	Seat vibration, pitch, and roll	Seat-weighted RMS reduced by 29.6%; pitch by 24.1%; roll by 26.7%	Full-scale road test	Preview sensors and edge computer	Road-tested; no agricultural field validation	[59]
Agricultural tractor seat	MR damper with ANFIS–ADRC	Seat acceleration and travel	Seat-acceleration RMS reduced by 68.9% vs. passive and 34.4% vs. PID.	Damper data and system simulation	Moderate training and tuning	No controller hardware or field validation	[52]
Agricultural tractor seat	MR damper with LADRC	Seat acceleration under random/ impact inputs	Seat-acceleration RMS reduced by 15.0% vs. PID and 24.2% vs. passive	Damper bench and system simulation	Low–moderate computation	Hardware timing and field tests not reported	[53]
Wide spray boom	Active trapezoid suspension with FPID	Boom roll and height tracking	Maximum chassis roll: 3.896°; boom inclination: 0.453°	6-DOF platform and field tests	Hydraulic actuation and multi-sensor integration	24 m prototype; application-specific setup	[117]
Planter/ harvester vibration diagnosis	Field vibration analysis with low-pass filtering and EEMD processing	Row-unit and cab vibration sources	Planter vibration concentrated at 10–50 Hz; key harvester excitation frequencies identified	Field and measured-signal studies	Multi-point sensing and signal processing	Diagnostic evidence; closed-loop vibration reduction not validated	[112,118]

**Table 6:** Multi-objective evaluation indices for agricultural semi-active suspension.

Category	Main Indices	Function	Limitation	Ref.
Occupant comfort evaluation	Body/seat acceleration RMS; frequency-weighted acceleration; WBV exposure	Operator comfort and WBV exposure	Does not reflect safety or operational accuracy	[3,133]
Chassis safety evaluation	Load transfer ratio; roll angle/rate; wheel lift-off; rollover threshold; tire/track contact stability	Chassis stability and rollover safety	Requires multi-sensor field tests	[128]
Operational accuracy evaluation	Boom-height error; seeding qualification rate; spraying deviation; header loss; trajectory/attitude error	Contribution to operational accuracy	Implement-specific metrics; difficult standardization	[114,116,117]
Energy and reliability evaluation	Control power; regeneration efficiency; force lag; control delay; actuation frequency; response stability	Deployment feasibility and service reliability	Requires long-cycle and multi-environment validation	[15,134,135]

**Table 7:** Validation hierarchy for agricultural semi-active suspension.

Validation Level	Validation Object	Function	Limitation	Ref.
Numerical simulation	Quarter-car, half-car, full-vehicle, random-road and farm-road models	Low-cost algorithm screening	Idealized; weakly reflects field environments	[37,41]
High-fidelity simulation	Multibody dynamics; finite elements; DEM-FMBD; TPA	Structural modes, contact boundaries, stress concentration, and vibration paths	High computational cost; unsuitable for online control	[42,44,48]
Bench and HiL validation	Damper bench; road simulator; HiL; human–machine or slip validation	Real-time hardware and actuator-response validation	Depends on model accuracy; cannot replace field tests	[58,136]
Agricultural field validation	Farm roads; slopes; soft soil; complex roads; chassis–implement tests	Real-condition engineering performance	Costly and condition-dependent	[50,92]

**Table 8:** Representative sensing and communication failures and fault-tolerant responses for agricultural semi-active suspension.

Sensor/ Communication Element	Failure Mode/Environmental Limitation	Control Consequence	Detection and Fusion	Degraded Operation/Fallback	Ref.
Camera/VIO	Illumination variation, crop/canopy occlusion, and sparse visual features	Reduced localization and preview confidence	Camera–IMU fusion and feature-quality monitoring	Disable preview; retain feedback control; vehicle-level safe stop if confidence remains low	[104,145]
LiDAR/look-ahead sensor	Noise, mounting/range trade-offs, and uncertain field recognition	Biased or noisy terrain preview	Camera–IMU cross-check, calibration, and plausibility monitoring	Observer-based road estimation or feedback-only control	[6,145,146]
IMU/INS	Bias and accumulated drift	State-estimation drift	Camera–IMU bias estimation; GNSS correction when available	Observer-based feedback and periodic bias correction	[104,145]
Suspension-travel/chassis-angle sensor	Ingress/contamination; drift; linkage/connector fault; saturation or stuck output	Biased state; incorrect damping/ leveling; missed end-stop/rollover warning	Range/rate/ stuck checks; IMU/observer residuals; redundancy if safety-critical	Observer state; fixed damping/passive mode; disable leveling/preview; safe stop if uncertain	[117,145]
GNSS	Signal degradation under dense foliage	Position/ velocity discontinuity	VIO/GNSS consistency monitoring and multi-sensor fusion	VIO/local-odometry mode; driver alert or vehicle-level safe stop if uncertainty persists	[104,145]
Edge communication	Terrain obstruction, soil-dependent propagation, and vibration-induced link degradation	Packet loss and delayed state/control updates	Link-health, latency, and packet-loss monitoring	Local edge processing and a cloud-independent safe controller	[147]

**Note:** The suspension-travel/chassis-angle sensor row combines reported sensor use with author synthesis. Inclination and cylinder-displacement sensing have been reported for boom control, whereas dedicated fault-injection and fault-tolerant field validation have not. The listed failure modes, checks, and fallback actions are, therefore, evidence-informed rather than directly validated.

**Table 9:** Evidence-bounded deployment routes for agricultural semi-active suspension.

Route	Evidence Basis	Online Burden	Communication	Deployment/Fail-Safe	Ref.
Reduced-order/invertible model	PI/algebraic models; processor timing and WCET: NR	Low; author synthesis	None for local execution	Local ECU; retain conventional damping if model confidence degrades	[21,23]
Explicit MPC	Offline explicit solution; Jetson-deployed ATV road test; WCET: NR	Low online; high offline	None for local execution	Local execution; fallback not reported	[59]
Offline-trained/ compressed learning control	DRL evidence mainly simulation-based; compression, timing, memory, and field validation: NR	Potentially moderate after compression; author synthesis	None for inference; intermittent for updates	Safety supervision and conventional fallback recommended; field validation: NR	[2,60]
Cloud–edge control	Agricultural edge architectures reported; suspension closed-loop validation: NR	Lower local burden; author synthesis	High	Safety-critical damping remains locally executable during link degradation	[147]

**Note**: NR, not reported; WCET, worst-case execution time. Evidence basis contains source-reported information; Online burden, Communication, and Deployment/fail-safe represent author synthesis unless otherwise stated. Compression and cloud–edge routes remain prospective because processor timing, memory use, and agricultural field validation are largely unreported. Safety-critical damping should remain locally executable.

## Data Availability

No new data were created or analyzed in this study. Data sharing is not applicable to this article.
